# Supplementary material for: Identification of the Genes Involved in Anthocyanin Biosynthesis and Accumulation in Taxus chinensis
Source: Genes (Basel). 2019 Nov 28;10(12):982. doi: 10.3390/genes10120982 (PMC6947853; doi:10.3390/genes10120982)
Supplement: Supplementary file 1 [file genes-10-00982-s001.pdf]

Table S1. Primers used for qRT-PCR

Gene name	Primer name	Sequence (5' to 3')
TcCHS	TcCHS-F	GGCCACCAGACACATTCTGA
	TcCHS-R	AAGAGAACGCCCATGTCCAG
TcCHI1	TcCHI1-F	AAGGCAGCTTTCAAGGACCA
	TcCHI1-R	GCTGTAGCCTTCTCTGGCAT
TcCHI2	TcCHI2-F	AAATGCAGTGAGAGACCGCC
	TcCHI2-R	TGGCAGGGAAATGGTAGGTG
TcF3H1	TcF3H1-F	GCCAGACATGACTCTGGGAC
	TcF3H1-R	CCGTCATCCTTGGTAGCCTG
TcF3H2	TcF3H2-F	CCCTGATTTGACACTGGGCT
	TcF3H2-R	CCCAGGATTTGCCATCGTCT
TcF3H3	TcF3H3-F	GGAATTAATCCAGCGCCTGC
	TcF3H3-R	TATCCCTCCAAAACACCCGC
TcF3H4	TcF3H4-F	CGAGATGTCCACAGCCCAAT
	TcF3H4-R	ATCCATCGACCATCCTTGCG
TcF3H5	TcF3H5-F	TTCTGTGCGACCAAGTTCCA
	TcF3H5-R	CCTCTGTGCTCGATGCTCTT
TcANS	TcANS-F	CACCACGGCAAATGGATCAC
	TcANS-R	TGTTCACCAGTCCTCTGTGC
TcF3'H1	TcF3'H1-F	GCAGCAGCATGGATGTGAAG
	TcF3'H1-R	CATGGATGAGGTTGGCGAGA
TcF3'H2	TcF3'H2-F	CAACGTACACCACTGCCTCT
	TcF3'H2-R	CATTGCAGAGGAGGACACCA
TcF3'H3	TcF3'H3-F	CCGATTCTCACGGACAGCTT
	TcF3'H3-R	CATTACGTGAGGATGGCGGA
TcF3'H4	TcF3'H4-F	GAATGTCTGCACCGCGAATG
	TcF3'H4-R	GCCAGCACCATCAGCTCTAT
TcF3'5'H	TcF3'5'H-F	AGTGGTTGGTCGTGATAGGC
	TcF3'5'H-R	GGTAGGCTGAGAGGAGTGGA
TcDFR1	TcDFR1-F	CAACTGATGGACCCTGGCAT
	TcDFR1-R	AGGCCGAAACAGAAGAGGTG
TcDFR2	TcDFR2-F	TCAGAGAAGACAGCATGGGC
	TcDFR2-R	GCAATGGTGGATCCTGAGGT
TcDFR3	TcDFR3-F	GCAGCCTGTGGCATTTTCAA
	TcDFR3-R	AAGACAACACTGGATGGCCC
TcDFR4	TcDFR4-F	GGTATGCGACTGCCAAGACA
	TcDFR4-R	AGGAGGTGTGGCTGTAAACC
TcDFR5	TcDFR5-F	TTCCACACAGCATGTCCAGT
	TcDFR5-R	CACGCTTCACCTTAGCCACT
TcDFR6	TcDFR6-F	ACTGTGAGACGGGTGATCCA
	TcDFR6-R	TCCACAGGAGTCCAGCAAGA
TcDFR7	TcDFR7-F	GAGGGGACGCTTGACATTCT
	TcDFR7-R	GCAGGATTCGTCAAGGAGGT
TcDFR8	TcDFR8-F	TGCACGTAACAGCCTTCGAT
	TcDFR8-R	TTCAACGCTGTCATCCACGA
TcANR	TcANR-F	TTGCTGGTTGTGACGGAGTT
	TcANR-R	GGTACAAGCCTTCAGAGCGT
TcLAR1	TcLAR1-F	GCGAATGTGATGCCAGCAAG
	TcLAR1-R	GTTGGCATGCCTCCTCATCC
TcLAR2	TcLAR2-F	CGACCCACGAACTCTCAACA
	TcLAR2-R	TCGGTAATGACTGCCCTTGG

Table S2. The proteins used in the sequence alignmentof CHS and CHI

Protein name	Plant Species	Accession number
AtCHS	*Arabidopsis thaliana*	NP_196897.1
VvCHS	*Vitis vinifera*	NP_001267879.1
OsCHS	*Ornithogalum saundersiae*	AIY34670.1
PcCHS	*Polygonum cuspidatum*	ABK92282.2
GmCHS	*Glycine max*	NP_001347353.1
DcCHS	*Dracaena cambodiana*	AIM54369.1
GbCHS	*Ginkgo biloba*	AAT68477.1
PsCHS	*Picea sitchensis*	AEN84253.1
PisCHS	*Pinus strobus*	CAA06077.1
PeCHS	*Phyllanthus emblica*	AHA61354.1
MsCHS1	*Medicago sativa*	AAA02823.1
MsCHS2	*Medicago sativa*	AAA02824.1
MsCHI	*Medicago sativa*	AAB41524.1
AtCHI1	*Arabidopsis thaliana*	NP_191072.1
AtCHI2	*Arabidopsis thaliana*	NP_201423.2
AtFAP1	*Arabidopsis thaliana*	NP_567140.1

Table S3. The proteins used in the sequence alignmentof F3H and ANS

**Protein name**	**Plant Species**	**Accession number**
GbF3H	*Ginkgo biloba*	ACY00393.1
AcF3H	*Aethusa cynapium*	ABG78792.1
AtF3H	*Arabidopsis thaliana*	NP_190692.1
GmANS	*Glycine max*	NP_001239794.1
VvANS	*Vitis vinifera*	ABV82967.1
ThcANS	*Theobroma cacao*	ADD51355.1
BjANS	*Brassica juncea*	ACH58398.1
AtANS	*Arabidopsis thaliana*	AEI99590.1
ApANS	*Acer palmatum*	AWN08246.1
CsANS	*Citrus sinensis*	NP_001275784.1
CasANS	*Camellia sinensis*	ALF36156.1
IbANS	*Ipomoea batatas*	ACT31916.1
MsANS	*Magnolia sprengeri*	AHU88620.1

Table S4. The proteins used in the sequence alignmentof F3’H and F3’5’H

Protein name	Plant Species	Accession number
VvF3'H	*Vitis vinifera*	BAE47006.1
DcF3'H	*Dracaena cambodiana*	AYM47547.1
AcF3’H	*Actinidia chinensis*	ADC34701.1
CsF3’H	*Camellia sinensis*	ALO62102.1
IhF3'H	*Iris x hollandica*	AHL83556.1
GmF3’5’H	*Glycine max*	NP_001236632.2
EsF3’5’H	*Epimedium sagittatum*	ADE80942.1
PaF3’5’H	*Petunia axillaris*	AUI38393.1
SmF3’5’H	*Salvia miltiorrhiza*	AWX67419.1
NmF3’5’H	*Nemophila menziesii*	BBA68555.1
CsF3’5’H	*Camellia sinensis*	ASU87427.1

Table S5. The proteins used in the sequence alignment of DFR and ANR

**Protein name**	**Plant Species**	**Accession number**
AtDFR	*Arabidopsis thaliana*	NP_199094.1
MnDFR	*Morus notabilis*	AHY35315.1
VvDFR	*Vitis vinifera*	NP_001268144.1
GaDFR	*Gossypium arboreum*	KHG11742.1
GbDFR	*Ginkgo biloba*	AGR34043.1
FtANR	*Fagopyrum tataricum*	AHA14497.1
VvANR	*Vitis vinifera*	BAD89742.1
RrANR	*Rosa rugosa*	AJO70135.1
RiANR	*Rubus idaeus*	AMP19723.1
GaANR	*Gossypium arboreum*	NP_001316937.1
MdANR	*Malus domestica*	NP_001280930.1
CsANR	*Camellia sinensis*	AHJ11240.1
MiANR	*Mangifera indica*	AIY25006.1

Table S6. The proteins used in the sequence alignmentof LAR

Protein name	Plant Species	Accession number
VvLAR1	*Vitis vinifera*	NP_001267887.1
VvLAR2	*Vitis vinifera*	NP_001268089.1
FtLAR	*Fagopyrum tataricum*	AHA14498.1
PaLAR1	*Picea abies*	AHB89627.1
PaLAR4	*Picea abies*	AIA08662.1
MrLAR	*Morella rubra*	AIX02997.1
PtLAR	*Pinus taeda*	CAI56321.1
CsLAR	*Camellia sinensis*	AZJ17294.1
CasLAR	*Camellia sinensis*	ASU87431.1

Table S7. Anthocyanin biosynthesis genes identified in different plant species

Enzemy	*Taxus chinensis*	*Arabidopsis thaliana*	*Salvia miltiorrhiza*	*Oryza sativa*
Chalcone synthase	*TcCHS*	*AtCHS*	*SmCHS1 SmCHS2 SmCHS3SmCHS4 SmCHS5 SmCHS6 SmCHS7 SmCHS8*	*OsCHS1 OsCHS2*
chalcone isomerase	*TcCHI1 TcCHI2*	*AtCHI1 AtCHI2 AtFAP1 AtCHIL*	*SmCHI1 SmCHI2 SmCHI3 SmCHI4*	*OsCHI*
Flavanone 3-hydroxylase	*TcF3H1TcF3H2 TcF3H3 TcF3H4 TcF3H5*	*AtF3H*	*SmF3H1 SmF3H2*	*OsF3H*
dihydroflavonol 4-reductase	*TcDFR1TcDFR2 TcDFR3 TcDFR4 TcDFR5 TcDFR6 TcDFR7 TcDFR8*	*AtDFR*	*SmDFR*	*OsDFR*
flavonoid 3’-hydroxylase	*TcF3’H1 TcF3’H2 TcF3’H3 TcF3’H4*	*AtF3’H*	*SmF3’H1 SmF3’H2 SmF3’H3 SmF3’H4 SmF3’H5 SmF3’H6*	*OsF3’H*
flavonoid 3’,5’-hydroxylase	*TcF3’5’H*		*SmF3’5’H*	
anthocyanidin synthase	*TcANS*	*AtANS1 AtANS2*	*SmANS*	*OsANS1 OsANS2*
anthocyanidin reductase	*TcANR*	*AtANR*		*OsANR*
leucoanthocyanidin reductase	*TcLAR1 TcLAR2*			*OsLAR*

Table S8. Chromatographic data of anthocyanin content determination

Sample Name[1]	Peak Area	Sample Concentration(ug/ml)[2]	Anthocyanin Content(ug/g)[3]
1-1Y	702913.750	7.53632625	301.45305
1-2Y	11433.485	0.35313813	14.1255252
1-3Y	20328.779	1.046491968	41.85967872
1-4Y	240211.656	3.065610378	122.6244151
2-1Y	1256054.375	4.2174825	168.6993
2-2Y	58856.355	0.06860091	2.7440364
2-3Y	174415.328	0.121972674	4.87890696
2-4Y	510935.063	1.441269936	57.65079744
3-1Y	819993.688	4.919962128	196.7984851
3-2Y	328665.500	1.971993	78.87972
3-3Y	252076.813	1.512460878	60.49843512
3-4Y	162804.844	0.976829064	39.07316256

[1]1Y to 4Y represent one to four years old xylem of *T. chinensis*. 1-1Y to 3-4Y represent three biological replicates.
[2]Sample Concentration was canculated according to the standard curve formula: y=6E-06x.
[3]Anthocyanin Content was canculated according to the dissolved volume and sample weight which were showed in the text.

Table S9. CDS and AA sequences of Anthocyanin Biosynthetic Enzyme Genes in *Taxus chinensis*.

Gene name	cDNA sequence	AA sequence
	ATGGCAGCAGGGAAGACGGTGGATCTGGAAG	MAAGKTVDLE
	CATTCAGGAAGGCGCAGAGGGCCGACGGCCC	AFRKAQRADG
	TGCCGCCGTCCTCGCCATCGGCACCGCCACTC	PAAVLAIGTAT
	CTCCCAACGCCATCGAACAGAGCTCCTACCCT	PPNAIEQSSYP
	GATTATTATTTCAGAATCACCAACAGCGAACA	DYYFRITNSEH
	CAAGGCCGAGCTCAAGGAGAAGTTCAAGCGC	KAELKEKFKR
	ATGTGCGAGAAGTCGATGATAAGGAAGAGGT	MCEKSMIRKR
	ACATGTATTTGACGGAGGAGATATTGAAGGAG	YMYLTEEILKE
	AACCCGAACGTGTGCGCGTACATGGCACCCTC	NPNVCAYMAP
	GTTAGACGCTCGGCAGGACATGGTGGTGGTGG	SLDARQDMVV
	AAGTGCCGAAGCTGGGGAAGGAAGCGGCTTC	VEVPKLGKEA
	AAAGGCCATAAAAGAATGGGGTCAGCCCAAG	ASKAIKEWGQ
	TCGAAGATCACACACCTCCTCTTCTGCACCAC	PKSKITHLLFCT
	CAGCGGCGTGGACATGCCCGGCGCAGACTACC	TSGVDMPGAD
	AACTCACTAAATTACTGGGACTCCGCCCTTCC	YQLTKLLGLRP
	GTGAAGAGAGTGATGATGTATCAACAGGGCT	SVKRVMMYQ
	GCTTCGCGGGCGGCACAGTTCTCCGCGTCGCC	QGCFAGGTVL
	AAAGACCTCGCAGAGAACAACCGCGGAGCCA	RVAKDLAENN
TcCHS	GAGTCCTGGTCGTCTGCAGCGAGATCACCGCC	RGARVLVVCS
	GTCACTTTCCGCGGCCCCTCCGACACCCACCT	EITAVTFRGPS
	CGACAGTCTCGTCGGCCAGGCTCTCTTCGGCG	DTHLDSLVGQ
	ACGGCGCCGCTGCCGTCATCGTCGGAGCGGAT	ALFGDGAAAVI
	CCCATTCCTGAGGTGGAGAAGCCCTCTTTCCA	VGADPIPEVEK
	ACTCCTCTTTACTGCTCAGACCATCCTTCCCGA	PSFQLLFTAQTI
	CAGCGAGGGCGCCATTGACGGGCACCTCAGA	LPDSEGAIDGH
	GAGGTTGGTCTCACCTTCCATCTTCTCAAAGA	LREVGLTFHLL
	CGTGCCCGGGTTGATCTCGAAGAACATCGAAA	KDVPGLISKNI
	AGGCGCTTGTGGAGGCCTTCCAGCAGTTCAAC	EKALVEAFQQF
	ATCTCCGACTGGAACGAGCTCTTCTGGATCGC	NISDWNELFWI
	CCACCCGGGAGGCCCTGCAATTCTTGACCAGG	AHPGGPAILDQ
	TGGAGTCCAAACTCCAACTGGACCCGAAGAA	VESKLQLDPKK
	GATGAGGGCCACCAGACACATTCTGAGCGAGT	MRATRHILSEY
	ATGGAAACATGTCGAGCGCCTGTGTGCTCTTC	GNMSSACVLFI
	ATTTTGGACGAGATGAGGAAGTCTTCCGCGGA	LDEMRKSSAD
	CAAGGGACATGCGACCACGGGAGAAGGACTG	KGHATTGEGL
	GACATGGGCGTTCTCTTCGGATTTGGTCCGGG	DMGVLFGFGP
	GCTCACCGTCGAGACCGTCGTTCTCAAGAGCG	GLTVETVVLKS
	TTCCCCTCGTCCATTAA	VPLVH*
	ATGGAGGTGGAAGGCGTCACATTCTCTTCTAA	MEVEGVTFSSN
	TGTTAGACCAGTTGGAAGCGACAAACAACTTA	VRPVGSDKQLI
	TCTTAGGAGGAGCAGGAGTTAGAGGGTTGGA	LGGAGVRGLE
	GGTAGATGGCAAGTTCATCAAATTTACAGCAA	VDGKFIKFTAI
	TTGGCATCTATTTGGAGGAGGACGTCCTTCCC	GIYLEEDVLPY
	TATCTCGCTCTCAAGTGGAAAAACAAGACGGC	LALKWKNKTA
TcCHI1	AGAGGAGTTGGGGACTGCCGAGGAGTTCTTCA	EELGTAEEFFM
	TGGATATTGTTACCTGTCCCTACGAGAAATAC	DIVTCPYEKYT
	ACAAGAGTGACACTCATCTTACCACTTTCTGG	RVTLILPLSGT
	CACCCAGTACTCTGAAAAAGTGTCTGAGGGCT	QYSEKVSEGC
	GCAAAGCGGCATGGGAAGCTGCCGGTATATAT	KAAWEAAGIY
	GGTGAAGCAGAGGCCCAGGCACTTGAACAGT	GEAEAQALEQ
	TTAAGGCGGTTTTTAACAACCACAATTTTCCG	FKAVFNNHNFP

	CCTGGGTCTTCCATTCAATTTACTCATTCACCT	PGSSIQFTHSPA
	GCCAGCCTTGTGATAGCATTCTCAAAGGACAG	SLVIAFSKDSLI
	TTTGATCCCTGAAAAGGCTGTAGATGTGATAG	PEKAVDVIENS
	AGAACAGCGTTCTATCCCAAGGGATTCTGGCC	VLSQGILASIIG
	TCTATCATTGGGAAAAATGGTGTATCTCCTCTT	KNGVSPLAKA
	GCTAAAGCTTCAATTGCAGAACGATTATCTAC	SIAERLSTLYFP
	ACTGTATTTTCCAGAGTTGGACCGTTTGGCTAT	ELDRLAILLSG
	TCTGCTTTCTGGAATGGAGGTGGATGGCATCT	MEVDGISFSAT
	CATTTTCAGCCACAATTCAACCCCCAGGATAT	IQPPGYKKEFL
	AAAAAGGAGTTCTTACTTGGAGGGGCTGGATT	LGGAGFRGLEI
	TCGAGGATTGGAGATCGAAGGCAAGTTCAGG	EGKFRKFTTIGI
	AAGTTTACAACAATTGGCATTTACATGGAGAA	YMEKSIISHLA
	GTCCATCATCTCTCATCTTGCCCTCACATGGAA	LTWKGKATKE
	AGGGAAGGCAACCAAGGAGTTGAACAATGCT	LNNALGFFMDI
	CTAGGTTTTTTCATGGACATTGTCACATGCCAC	VTCHYEKFAK
	TATGAAAAGTTTGCCAAGGTATCCATGATTGC	VSMIAPLSGTQ
	CCCACTAAGCGGTACTCAATACTCTGAGAAGG	YSEKVSENCQ
	TCAGTGAGAATTGCCAGGCAGCTTGGGAAGCT	AAWEAAGIYG
	GCAGGAATATATGGTGAAGCTGAGGCCAAGG	EAEAKALEAF
	CATTAGAAGCTTTTAAGGCAGCTTTCAAGGAC	KAAFKDQNFPP
	CAGAATTTTCCACCTGGTTCTTCCATCCTGTTT	GSSILFTVSPAG
	ACTGTTTCACCTGCAGGCCTTGTGATTGCGTTC	LVIAFTKDSSM
	ACCAAGGATAGTTCAATGCCAGAGAAGGCTA	PEKATAVIDNR
	CAGCAGTTATTGATAACAGAGTGCTTGGGGAA	VLGEAVLASMI
	GCAGTGCTGGCTTCTATGATTGGGGAAAATGG	GENGVSPSAK
	TGTTTCTCCATCGGCCAAAGCATCTTTAGCAG	ASLADRLSQLL
	ATCGACTCTCTCAGCTTCTGCAATAA	Q*
	ATGGCATCTGAACTTGTAATGGTGGATGAGAT	MASELVMVDE
	CGAATTCCCCAAGTCTGTTACGCCGTCTGCAT	IEFPKSVTPSAS
	CTTCTAAAACTTTAGGCATCATCGGACATGGA	SKTLGIIGHGIT
	ATCACCGACATAGAAATCCATTTCCTGCAAAT	DIEIHFLQIKFT
	CAAATTCACGGCCATTGGCGTCTACGTGGATG	AIGVYVDVDV
	TCGACGTGGCTTCGCATCTGCAGGCGTGGAAA	ASHLQAWKAK
	GCAAAGACTGCTGCTGAGCTCTTGGCAGACGA	TAAELLADDSF
	TTCATTCTTCGATGCCCTTTTGCAAGCTCCGGT	FDALLQAPVEK
	AGAGAAATTTATCAGAGTGGTGGTTATAAAGG	FIRVVVIKELK
	AGCTCAAGGGTTCACAATATGGTCTACAGTTG	GSQYGLQLEN
TcCHI2	GAAAATGCAGTGAGAGACCGCCTAGTGGCCA	AVRDRLVAID
	TTGACAAATATGAAGACGAGGAAGAGGAAGC	KYEDEEEALA
	GCTAGCTAAGGCTGTGGAGTTTTTCCAAGGCA	KAVEFFQGKY
	AATATTTGAAGAAGAATTCAGTCATCACCTAC	LKKNSVITYHF
	CATTTCCCTGCCAACGCCAAAACTGTTGAGAT	PANAKTVEMS
	GAGCTTCGTTACAGACGAGAACAAAGCGTAC	FVTDENKAYKI
	AAAATTAATGTAGAGAATGGAGATGTAAGCA	NVENGDVSSMI
	GCATGATAAAGAAGTGGTACCTTGGGGGCTCC	KKWYLGGSSA
	TCAGCTGTGTCCTCCTCAACAATTGCATCTCTT	VSSSTIASLAEG
	GCAGAAGGCATTGCTGCTCTGCTAAAATAG	IAALLK*
	ATGGCTCCCGCTGTAGAAGTGGTGGCTTCAGG	MAPAVEVVAS
	GCGACCCGACATACTTCCAGTCGGGGAAGCAG	GRPDILPVGEA
	AGGCGGCGAAGACGTTGCAGTCGATCTTCATC	EAAKTLQSIFIR
TcF3H1	CGAGACGAGGACGAGCGGCCCAAAGTGGCTT	DEDERPKVAY
	ACAACAACTTCAGCAAAGACATTCCCATCATT	NNFSKDIPIISL
	TCGCTGGAAGGAATTGAGGGGAAAGATAGAG	EGIEGKDRDRV
	ATCGAGTGAAGGAGGAAGTGAGCAATGCCTG	KEEVSNACKE

	CAAGGAGTGGGGATTGTTCCAGGTGGTGAATC	WGLFQVVNHG
	ATGGAGTGCCCAAGGAGCTGGTGCAGACCAT	VPKELVQTMN
	GAATCAGCTCGCCAGAGAATTTTTTGCGCTCC	QLAREFFALPA
	CTGCGGAGGAGAAGCTGAAATACGATATGCG	EEKLKYDMRG
	AGGAGGGAAGCGAGGTGGGTTTGTGGTCAGT	GKRGGFVVSS
	AGCCATCTTCAAGGCGAAGCCGTTCTGGACTG	HLQGEAVLDW
	GAGAGAAATCTGCACTTACTTCTCATATCCCC	REICTYFSYPL
	TGCATCAGCGTGATTACACCCGCTGGCCTGAA	HQRDYTRWPE
	AAGCCAGAGGGGTGGAGAGACGTTGTGGACA	KPEGWRDVVD
	AGTACAGCGGAGCCCTGATGAATCTGGCCTGC	KYSGALMNLA
	AAACTGCTGGAAGTGATCTCGGAGGCGCTGGG	CKLLEVISEAL
	ACTAGACGCGGAAGCCGTGACCAAGGCGTGC	GLDAEAVTKA
	GTGGAGATGGACCAGAAAGTTGTGATCAATTA	CVEMDQKVVI
	CTACCCAAAATGCCCTCAGCCAGACATGACTC	NYYPKCPQPD
	TGGGACTGAAAAGGCACACCGATCCGGGCAC	MTLGLKRHTD
	CATCACGATCCTCCTGCAAGACCACGTAGGAG	PGTITILLQDHV
	GCCTCCAGGCTACCAAGGATGACGGCCTAAAC	GGLQATKDDG
	TGGATCACCGTCGAGCCCGTCGAGGGAGCCTT	LNWITVEPVEG
	CGTCGTCAATCTCGGAGATCACATGCATTATC	AFVVNLGDHM
	TGAGCAACGGAAAATTCAAGAGCGCGGACCA	HYLSNGKFKS
	TCAGGCGGTGGTGAACTCGAACAGCACCAGG	ADHQAVVNSN
	CTGTCCATCGCGACGTTCCAGAATCCTGCGCA	STRLSIATFQNP
	GGATGGAATCGTTTACCCGCTGAAACTGGAGG	AQDGIVYPLKL
	AGGGTGAGCAATGCATGATGGAAGAGCCCAT	EEGEQCMMEE
	CACCTTCGCTCAGATGTATTCTCGCAAGATGG	PITFAQMYSRK
	GTCGGGACATCGAACTCGCTCGCCAGAAGAA	MGRDIELARQ
	GCTCGCCAAGGTCGGCGTTGAAGAAGCTCCGA	KKLAKVGVEE
	AATCCTGA	APKS*
	ATGGCACCTCCCTGTTTGGAAGAGAGCGTGAT	MAPPCLEESV
	GAGTTTAGCGGAGTCGGGGGTGAAAGAGCTG	MSLAESGVKE
	CCTAAAAGTTACTTAAAGGACGAGGATGAAC	LPKSYLKDEDE
	GCCCCACCGTTCCCCACAATGTTTTCTGCCAG	RPTVPHNVFCQ
	GACATCCCTGTCATATCCCTCCTCAATTCTCAC	DIPVISLLNSHT
	ACCCATGGCCCTGAAAAAGATAGACTAAAAG	HGPEKDRLKA
	CCCAGCTGAAAAAGGCCTGCCAAGAGTGGGG	QLKKACQEWG
	AATTTTTCAGATTGTAGATCACCGAGTCCCCA	IFQIVDHRVPK
	AGGATCTCACGACCCTCATCATGAGCGCCGCC	DLTTLIMSAAM
	ATGGATTTTTTTTCGCTACCTGCTGAGGAGAA	DFFSLPAEEKL
	ACTGCAGTACGCCCTGAAGCCAGGAAGCTATG	QYALKPGSYV
	TAGGGTATGGAAACGGAAGTTTTATGAGGGAC	GYGNGSFMRD
TcF3H2	GACCCACTCATGGATTGGAGAGAGTTGTATGT	DPLMDWRELY
	GACCAGATGTTTGCCTCGTGACATTACCCTCT	VTRCLPRDITL
	GGCCCTCCAACCCACCCTCTATGCGGAAAACT	WPSNPPSMRK
	ATTGCAGATTACAGTGACGCAACGCTGGCATT	TIADYSDATLA
	GGTAACAGAGTTGTTAGAGCTCATATCCGAGG	LVTELLELISEA
	CTCTGGGGCTGGAGTGTAAAGCTATTGAGAAT	LGLECKAIENA
	GCGTGTGGAGAGGAGGAGCAGAAGCTTCTGC	CGEEEQKLLLN
	TAAATTATTACCCCAAATGCCCTCGCCCTGATT	YYPKCPRPDLT
	TGACACTGGGCTTGAAGAGGCACACGGATCCG	LGLKRHTDPGT
	GGCACCATCACTCTGTTGCTCCAAGACAAAGT	ITLLLQDKVGG
	TGGCGGTCTCCAGGTCACTAGAGACGATGGCA	LQVTRDDGKS
	AATCCTGGGTCACCGTGCAACCAATCGAGGGG	WVTVQPIEGAF
	GCATTCGTAGTTAATTTGGGAGATCAGATGCA	VVNLGDQMHV
	TGTACTAAGTAACGGGATTTTGAAGAGCGCAG	LSNGILKSADH

	ATCATCAAGCTGTAGTGAATTCAAGCACAACG	QAVVNSSTTRL
	CGGTTGTCCGTAGTAACCTTTCACAATCCCAA	SVVTFHNPNPN
	CCCTAATTCCATGGTTTATCCGCTTGACGGCTT	SMVYPLDGLV
	GGTTGACGAGGAGCATCCCACCAAATTTGAAC	DEEHPTKFERY
	GCTACATTTACAAAGATTTCTACGGGAGAAAG	IYKDFYGRKM
	ATGACCCAACATGTTGCAGAGAGAGCCAAAA	TQHVAERAKK
	AAATGAGATTGAAGGAAGGGAAGAAGAGCAA	MRLKEGKKSN
	CACACCTCAACAAAAGTTTTAA	TPQQKF*
	ATGGCAGCAGTTCGAGTTCAATCCCTTGCAGG	MAAVRVQSLA
	AAGTGGAGTAACAAGTATACCAGCGGAATTC	GSGVTSIPAEF
	GTGAGGCCTCTCCACGAGAGGCCTAGTGCCAT	VRPLHERPSAI
	TGGCAATGACGGTCCTTCCGATCACCGTCTTC	GNDGPSDHRLP
	CCGTCATCGATTTGGCAAATTTAGATGTTTCCG	VIDLANLDVSG
	GCCATCTCTGTGAGAAAACCGTATCTGAAATA	HLCEKTVSEIG
	GGAAGCGCGGCTCAGCAATGGGGACTTTTCCA	SAAQQWGLFQ
	GATAGTGAATCACGGAATTTCTGAGGAATTAA	IVNHGISEELIQ
	TCCAGCGCCTGCAGGGTGTTGGCAAGGAATTC	RLQGVGKEFFE
	TTCGAAATTCCGCAGGAGGAAAAGGAGAAGT	IPQEEKEKYAN
	ACGCCAACAATGCGGCGGCGGGTGTTTTGGAG	NAAAGVLEGY
	GGATATGGCACCAAGTTGGCCCATAATATTGA	GTKLAHNIDG
	TGGAAAACTGGAGTGGATTGACTACTTTTTCC	KLEWIDYFFHI
	ATATACTCTGGCCTCCTTCCAAAAGGGACTTC	LWPPSKRDFKT
	AAAACCTGGCCTAACCACCCTCCCACTTACAG	WPNHPPTYRE
TcF3H3	AGAGGTGAACGATGAATATGGTAGGGCAATTT	VNDEYGRAILG
	TGGGTGTAATGAATAAGCTTCTCACGGCCCTT	VMNKLLTALSI
	TCTATCTCTCTCGGAGTGCAAGAATGTGGGCT	SLGVQECGLKE
	GAAAGAGGCACTTGGTGGTGAAGATTTAGAAT	ALGGEDLELE
	TGGAGATGAAAATAAATTACTACCCACCATGT	MKINYYPPCPQ
	CCCCAACCTGAGCTTGCTCTGGGAGTGGAACC	PELALGVEPHT
	CCACACAGACATGAGTGTTCTCACTCTTCTCGT	DMSVLTLLVS
	CTCCAATGAAGTTCCAGGTCTCCAAGTGCACA	NEVPGLQVHN
	ACAATGGCCGTTGGGTTACTGTAGATTATGTT	NGRWVTVDYV
	CCTAATGCTATCACCGTCCACATCGGAGACCA	PNAITVHIGDQ
	GCTGGAGGTTCTGAGCAATGGCAAGTACAAG	LEVLSNGKYKS
	AGTGTTTTGCACAGGAGTTTGGTGAGCAAGGA	VLHRSLVSKD
	CAAAGTGAGAATGTCATGGCCGATATTCTGCG	KVRMSWPIFC
	CTCCTCCTGTTGATAAAGTCATCGGCCCCATG	APPVDKVIGPM
	AAAGAATTAGTCAATGAAGACAATCCTCCGTT	KELVNEDNPPL
	GTACAATGCCAAGAGTTATGCAGAGTTCAAGC	YNAKSYAEFK
	ATCGTAAGATCAACAAGCTGAAGCAGTAG	HRKINKLKQ*
	ATGTGCAGGCAGCCTCAATCTCAACAAGTAAA	MCRQPQSQQV
	CGCCTTCTCCAAAGTTAACTCACCTCACAACA	NAFSKVNSPHN
	CTGGAGAGCTCGATCTGTCGTATTCGCGAGTT	TGELDLSYSRV
	TGGATGGCCCAATCAAGTCCCTCCGATAATGG	WMAQSSPSDN
	TAAGGGAGTGTTGTTGCAAGCAGAATGGCCGG	GKGVLLQAEW
	AGCCAATTCAGAGGGTTCAATCCCTCGCGGAA	PEPIQRVQSLA
TcF3H4	AGCGGTATTAACGTGGTGCCGCCCAGGTATAT	ESGINVVPPRYI
	TAAGGCGGAGAAGGACAGGCCTGCAGTAGAT	KAEKDRPAVD
	ATGGATAGAGAGAGAGATATAGATGATTCGCT	MDRERDIDDSL
	GGAAGTAAACATACCTATCATCGATCTCGGGG	EVNIPIIDLGGL
	GACTAGAGGGCGAGGGTCGAGAGGGTACGAT	EGEGREGTMQ
	GCAGCAGATCTGGATGGCGTGCAGGGAATGG	QIWMACREWG
	GGTTTCTTCCAGGTGCTCGGTCATGGCGTCCCT	FFQVLGHGVPL
	CTCCATCTCATACGCCAAGCCAGACAAGTTGC	HLIRQARQVAR

	TCGACAGTTTTTTAGTTTGCCTCTCGAAGAAA	QFFSLPLEEKQ
	AGCAATCGTATGCCAATTCCCCAAAGACGTAC	SYANSPKTYEG
	GAGGGATATGGCAGCCGACTCGGTATCGACA	YGSRLGIDKGA
	AGGGCGCTTTGCTCGACTGGGGAGACTATTTC	LLDWGDYFFL
	TTTTTGCACTTGTTGCCTCTCTCCATCAAAGAT	HLLPLSIKDINK
	ATCAATAAATGGCCTGCTAAGCCCACTTTGTA	WPAKPTLYRES
	CAGAGAAAGCATAGAGGAATACGGGAAGCAA	IEEYGKQVGKL
	GTAGGCAAGTTGTGCGAGATGCTGCTGGGTGT	CEMLLGVLSIN
	GTTGTCCATCAATGTGGGGTTGGAAGAGGGTT	VGLEEGYFEEA
	ACTTTGAAGAGGCTTTCGGGAGTGGGAGTGGG	FGSGSGSVGAC
	AGTGTTGGAGCTTGTATGAGGATGAATTATTA	MRMNYYPRCP
	CCCGAGATGTCCACAGCCCAATCTCACGTTGG	QPNLTLGLSSH
	GATTATCGTCGCACTCCGATCCAGGTGGCATG	SDPGGMTVLLP
	ACGGTGTTGCTTCCTGATGAGACGGTGCGGGG	DETVRGLQVR
	CCTACAGGTGCGCAAGGATGGTCGATGGATTT	KDGRWILVEP
	TAGTGGAGCCTCATCCGCACGCCTTGATCGTC	HPHALIVNIGD
	AACATTGGCGATCAACTTCAGATACTAAGCAA	QLQILSNGIYK
	CGGCATATACAAAAGCGTGGAGCACCGCGTG	SVEHRVVVNS
	GTTGTAAATTCGGAAAAAGACCGTGTGTCGAT	EKDRVSIALFY
	AGCACTATTTTACAACCCGGACGGAGACAAAA	NPDGDKIIQPA
	TAATCCAACCTGCAGGACAGCTGGTAAATGAA	GQLVNEESHSP
	GAGTCGCACTCGCCCCCCTTGTACCAGCCAAT	PLYQPMTFNEY
	GACATTTAATGAGTACAGATTATTCATACGTA	RLFIRKRGPLG
	AAAGAGGTCCCCTCGGAAAATATCAAGTAAAT	KYQVNSITNSA
	TCCATCACAAATTCAGCCTCGTCGTGA	SS*
	ATGTCTAGAAGACAACTTACTTGGCTTTTCTCT	MSRRQLTWLF
	GTAGGCGTAACAATGGAGAAGCAGGCTGTGG	SVGVTMEKQA
	CCGCCATTACCAGAGTTCAGACCTTGGCAGAG	VAAITRVQTLA
	AGCGGAAATCAAACTGTTCCGCCGCAGTATGC	ESGNQTVPPQY
	CAGATCCGGCCACCTAGAAACATTACAGAACC	ARSGHLETLQN
	CTTGTCACGCACGCAACATCCAGGTTCCTGTC	PCHARNIQVPV
	ATCGACTTACACGCCTTGAACGTCGAAAATCT	IDLHALNVENL
	GAGGGATAAAACCACCGCAGAAATTTCAAGC	RDKTTAEISSA
	GCTGCAGAAAATTGGGGCTTCTTTCAGATCAT	AENWGFFQIIN
	CAATCATGGAATCCCGGAGACTCTAATCGCTC	HGIPETLIARV
	GGGTGCAGGCAGCGGGAAGAACCTTCTTCGAC	QAAGRTFFDLP
	CTTCCAATCGAAGAAAAAGAGTTGTACAAAA	IEEKELYKNDG
	ATGACGGAGCAGGGAATCCGGTTGGCTATGGC	AGNPVGYGSK
	AGCAAGCTTGGATACACTGCGGATGCTAAATT	LGYTADAKLD
TcF3H5	GGACTGGGGAGATTACTTTTACAACGCTGCTT	WGDYFYNAA
	GGCCTCCTGCCGCGAGAGATCTGGCGAAATGG	WPPAARDLAK
	CCGAAGCAGCCTTCGGATTTCACGGAAGTGAT	WPKQPSDFTEV
	GGATGAATACGGCAGAGAGGTTTACAAATTGT	MDEYGREVYK
	GGGAAGTGCTTATGCAGGCGCTTTCTAGAGGA	LWEVLMQALS
	CTAGGACTGGAGGATGAAAATAGGCTGAGCG	RGLGLEDENRL
	AGAGGATCGGAGGAGAGGGAAAGGAAATCCA	SERIGGEGKEI
	TTTCAGGATTAACTACTATCCGCCATGTCCTCA	HFRINYYPPCP
	GCCGGAGCTGGTTTTGGGGCTTTCACCGCACT	QPELVLGLSPH
	CTGATCCAAACGCCCTCACAATTCTTCTGTGC	SDPNALTILLC
	GACCAAGTTCCAGGCCTGCAAATTCGCAAAGA	DQVPGLQIRKD
	CGGCGCTTGGTTTCATGTCCAGACCGTTCCAG	GAWFHVQTVP
	GCGCTCTCGTTGTCAACATCGCGGACGCGCTG	GALVVNIADA
	GAGGTAGTAAGCAATGGAAAATACAAGAGCA	LEVVSNGKYK
	TCGAGCACAGAGGCATGGTGCACAAGGATCG	SIEHRGMVHK

	GACAAGAATGTCGTGGGCAATGTTCTGTGCAC	DRTRMSWAMF
	CGAAGCGTGAAGTGATCATCTCGCCTCTTGAA	CAPKREVIISPL
	GAATTGTTAAACCAAGGAAATCCTGCATTATA	EELLNQGNPAL
	CGGGGCCTCCTCTTATGAAGAACATCTGCAAA	YGASSYEEHLQ
	AGCTTTTTACCAAAGGAGTCGGTGGGAAGACG	KLFTKGVGGK
	TACGTACATCAGCTCAAACAATTATAG	TYVHQLKQL*
	ATGGCGCCGGCTCGAGTGGAAACCCTGTCGGG	MAPARVETLS
	CAGCGGCCTGGAATCCATTCCTCTCGAGTACG	GSGLESIPLEY
	TGAGACCCGTTGAAGAGAGGCCGACCGACAG	VRPVEERPTDS
	TGTTCTGGATCTCACGGACCATGGCCCTCAAC	VLDLTDHGPQ
	TTCCCGTCATCGACTTCGCCGGCTGGCATTTCG	LPVIDFAGWHF
	GCGACGCGGAGGAGAGGAAGAAGATCATGCG	GDAEERKKIM
	GGAAATAGCCGAGGCGAGCCAGGAATGGGGA	REIAEASQEWG
	GCCATGCAGCTGCTCAACCACGGCATCTCTCA	AMQLLNHGIS
	ATCTCTCATTGCTCGTCTCCAGGCCGCGGGAA	QSLIARLQAAG
	AGGCCTTCTTCGACCTCCCCATCGAAGACAAG	KAFFDLPIEDK
	GAAAAATACGCCAACGACTCGGGCAGCGGGA	EKYANDSGSG
	AGATCTTGGGCTACGGAAGCAAGCTTGCCAAC	KILGYGSKLAN
	AACGCGAGCGGGCAGCTGGAATGGGAGGACT	NASGQLEWED
	ACTACTTCCACATTCTTTGGCCCGACCACAAG	YYFHILWPDH
	CGCGACGTGGCCAACACGTGGCCCGGCTATCC	KRDVANTWPG
	TTCAGACTACGAGGAGGTCACCGTCGCCTACG	YPSDYEEVTVA
	GTCGCGAGATCCGCAAGCTCACCGCTCAGATT	YGREIRKLTAQ
TcANS	CTGGGCGCGTTGTCGTTAGAGTTGGGGTTGGA	ILGALSLELGL
	GGAGGGTCGCATGGAGCGGGTGCTGGGCGGC	EEGRMERVLG
	GAGGAGCTACTGATGGAGTTGAAGATCAATTA	GEELLMELKIN
	CTATCCGCGGTGCCCTCAACCCGAGCTGGCGT	YYPRCPQPELA
	TGGGTGTGGAAGCGCACACCGACATCAGCGCC	LGVEAHTDISA
	CTCACATTCCTTCTCCACAATATGGTGCCGGG	LTFLLHNMVP
	CCTCCAGCTCTTCCACCACGGCAAATGGATCA	GLQLFHHGKW
	CCGCCAAATGCATTCCCGGGGCCCTCATTCTT	ITAKCIPGALIL
	CAGCTCGGCGACCAAGTTGAGATAATGAGCA	QLGDQVEIMS
	ACGGGAAATACAGTAGCGGGCTGCACAGAGG	NGKYSSGLHR
	ACTGGTGAACAAGGAGAAAGTGAGGATATCG	GLVNKEKVRIS
	TGGGCGGTCTTCTGCTGCCCTCCCAAAGACGC	WAVFCCPPKD
	CGTAATCGGGCCCATGAAAGAAATGGTGGAC	AVIGPMKEMV
	GACCAAAACCCTCCGCTCTTCCCAGCCAGGAC	DDQNPPLFPAR
	TTTCCAGGAACACATCGACTACAAACTCTTCA	TFQEHIDYKLF
	GGAAGAACCGGGCCAAAGAAAAAGCTACCAA	RKNRAKEKAT
	CTGA	N*
	ATGGAGATGAATTTGAATGTGTTGGGTTATGT	MEMNLNVLGY
	TGTATTGGGCATCGTATCAGTTTATGCGATTTA	VVLGIVSVYAI
	CAGGAGATTTTGTCCGGGGATGAAATTACCGC	YRRFCPGMKL
	CAGGTCCTGGCGAGTGGCCTGTAATTGGGAGC	PPGPGEWPVIG
	CTGCATCTTCTAGGCAGTCTGCCCCACAGATC	SLHLLGSLPHR
	CCTGGATCGACTCTCCCAGAGGTATGGCCCAA	SLDRLSQRYGP
	TCATGTATGTCAAGCTGGGCTCCGTGCCCTGC	IMYVKLGSVPC
TcF3'H1	GTTGTTGCATCCTCCGCCGACATGGCGCGCGA	VVASSADMAR
	GTTTCTGAAGACGCACGACCTTACTTTCTCCTC	EFLKTHDLTFS
	TCGCCCCAAGATCGCCGCCGGAAAGTACACTG	SRPKIAAGKYT
	TGTACAATTACTCCGATATAACGTGGTCTCCCT	VYNYSDITWSP
	ACGGAGATCACTGGCGGCGGGCCAGAAAAAT	YGDHWRRARK
	ATGTTTGATGGAGTTGTTCAGCAAGAAGCGGT	ICLMELFSKKR
	TGGAGTCGTTCGAATACATTAGAATGGAAGAG	LESFEYIRMEE

	GTTTGCAGCATGGTGGCCTCTGTTTTCCGAACC	VCSMVASVFR
	TCCGCCGGCGGTCTCCCCGTCCATCTGAGAGA	TSAGGLPVHLR
	GGAGACGTACACCGTGAGCAATAATATTATCT	EETYTVSNNIIS
	CTCGAATGGTGCTGGGGCGGAGATATCTGGAA	RMVLGRRYLE
	GAGAGCGCGAATCACAAGATAAAACCGCATG	ESANHKIKPHE
	AATTCAAGGAGATGCTGCAGGAGCTGTTCGTG	FKEMLQELFVL
	CTCAACGGCGTGTTTAACATTGGAGACTATCT	NGVFNIGDYLP
	GCCCTGGCTCGGCTTCCTCGATCTGCAGGGGT	WLGFLDLQGY
	ATGTGAAAAGAATGAAGGCGCTGAGCAAACG	VKRMKALSKR
	GTTGAATGTTTTTCTGGAGGAAGTGTTGGAAG	LNVFLEEVLEE
	AACACGAGAGGAGGAGGAGGAGCGTGCCCGA	HERRRRSVPDY
	TTATGTCCCCGCCGACATGGTCGATGTTTTGCT	VPADMVDVLL
	GCAACAGAGCGATGATCCCAACAACCACCTCT	QQSDDPNNHL
	CCCCAAACAGAGTCAAAGGCTTCACTCAGGAC	SPNRVKGFTQD
	ATGATAGCCGGCGGAACAGAGAGCTCAGCAA	MIAGGTESSAT
	CGCTTGTGGAGTGGGGACTCGCGGAGCTCCTA	LVEWGLAELL
	AAAAAGCCGGAAATCTTCGCCAAGGCCAGCG	KKPEIFAKASE
	AAGAAATGGAGAGAATTGTTGGAAAGGAAAG	EMERIVGKER
	ATGGGTGGAAGAGAAAGACATCGCCAGCATG	WVEEKDIASM
	GAGTATCTGCAATGCATAGTGAAAGAAACGAT	EYLQCIVKETM
	GAGGCTGCACCCGGTCGCCCCTCTCTTGGTTC	RLHPVAPLLVP
	CCCATCTCTCCACCGAGCACTGCAAAATTGCA	HLSTEHCKIAG
	GGCTACGACATTCCTGCCAACACCAGATTATT	YDIPANTRLFV
	CGTGAGCGTGTGGACCATCGCAAGAGACGAG	SVWTIARDEQL
	CAACTGTGGGAGAAAGCTGAGGAATTCAGGC	WEKAEEFRPER
	CAGAGAGGTTCCATGGCAGCAGCATGGATGTG	FHGSSMDVKG
	AAGGGCACAAATTTTGAGCTTCTTCCCTTCGG	TNFELLPFGAG
	TGCAGGGCGAAGAATGTGCCCTGGCTATAATC	RRMCPGYNLG
	TGGGTCTCAAAGTGGTTCAGCTGGGTCTCGCC	LKVVQLGLAN
	AACCTCATCCATGGATTCCACTGGCGCCTTCC	LIHGFHWRLPP
	ACCTCATCAAGAATTAGACATGACAGAGACCT	HQELDMTETY
	ATGGCCTTTCTACACCCAAAGCAGATCCTCTC	GLSTPKADPLV
	GTAGCCATGGCGGAGCCTCGCCTGCCTTCCCA	AMAEPRLPSHL
	TCTCTACAGTTTCACTTAA	YSFT*
	ATGGATTTAAGCATGAGCCACAGCATATGGTA	MDLSMSHSIW
	CTACTATTCTATCACCCTTATTATAGCACTTAT	YYYSITLIIALII
	TATAGCGGGTAGAAAATGGATGAGCGCCGCC	AGRKWMSAAT
	ACTTCCAGGCTTCCTCCGGGACCTTTCGCCCTG	SRLPPGPFALPL
	CCCCTGTTCGGCCATCTCCATCTCCTGCAACCC	FGHLHLLQPNV
	AACGTACACCACTGCCTCTCCGAGCTCTCCCA	HHCLSELSHKY
	CAAATACGGAGCTCTCATGTCCTTCAAATTCG	GALMSFKFGL
	GATTGAAAACATCAATCGTGGTGTCCTCCTCT	KTSIVVSSSAM
	GCAATGGCGAAAGAAATCCTGAAAGAAAACG	AKEILKENDQS
TcF3'H2	ACCAGTCTTTTGCAAACAGAAGCGTACCAGTG	FANRSVPVVA
	GTGGCCAGATGCATCGCATACAATGCTTCGGA	RCIAYNASDIL
	CATTCTGTGGAGTCCCTACGGCCCCAGATGGC	WSPYGPRWRL
	GCCTCCTCAGAAAAATCTGCGTCAAGGAGCTT	LRKICVKELFS
	TTCAGCCCCAAAAGCACGGAATCCCTGCGCCC	PKSTESLRPLR
	TCTGAGAAAAGAAGAGGTCAGAAGAACAGTG	KEEVRRTVGNI
	GGAAACATTTACAGGGACTCCATTAATGGGGT	YRDSINGVSVD
	TTCTGTGGATGTGGGTGCCCAGGCTTTTGTGA	VGAQAFVTSL
	CTTCGCTGAATCTTATTACAAATATGATGTGG	NLITNMMWST
	AGCACAAATTCTGAGACGGGTGAAAGGGGGG	NSETGERGVEF
	TGGAATTTAAGGAGCTGATGGGAGAACTGGTT	KELMGELVYV

	TATGTTCTGGGTGTGCCCAATGTTTCGGATCTG	LGVPNVSDLFP
	TTTCCCTTTCTGGAGAGACTGGATGTGCAGGG	FLERLDVQGLY
	ACTTTACAGGAGGATGCACAAGGTTTTTCTCA	RRMHKVFLRF
	GATTTGATAGGCTCTTTGATGGTATTATTGAG	DRLFDGIIEERV
	GAGAGGGTGAAGGGTACAAGTAAGGGAAATG	KGTSKGNDFL
	ATTTTCTCCAGTCTTTGCTTGATCTGACAGAAA	QSLLDLTERGV
	GAGGTGTGGATGAAGACGATCCTGAGAGTGTT	DEDDPESVQIT
	CAGATCACCATGAATGATATTAAAGTTCTGTT	MNDIKVLLMD
	GATGGACATGGTAACTGGATCAACTGATACAA	MVTGSTDTTS
	CATCCAATACATTGGAGTGGGCAATGGCAGAG	NTLEWAMAEL
	CTCATACAAAAACCAGACATAATGAAGAGAG	IQKPDIMKRAQ
	CCCAAAAAGAAATAGAAGAAGTTGTGGGGTT	KEIEEVVGLDH
	GGACCATATTGTAGAAGAGTCTCACCTATCCC	IVEESHLSQLP
	AACTACCATACTTAGACATAATAGTAAAGGAA	YLDIIVKEVLR
	GTACTAAGGCTGCACCCTGCACTACCTCTGCT	LHPALPLLAPH
	TGCACCCCACTGTGCAGACAAAACCTGTGAAA	CADKTCEIGGY
	TTGGAGGGTACATCATTCCAAAGGATACTCAA	IIPKDTQVLVN
	GTCCTGGTTAATGTGTGGAGTATTCAAAGGAA	VWSIQRNPQV
	TCCACAAGTGTGGAAGGATCCATCAGTGTTCA	WKDPSVFNPE
	ATCCAGAGAGGTTTTTGGATTCAAAGTGGGAT	RFLDSKWDYN
	TACAATGGGAGAGAGTTTGATTATTTCCCATT	GREFDYFPFGS
	TGGGTCAGGTAGAAGGATATGTGCAGGGCTTT	GRRICAGLSMA
	CCATGGCCACTAGAATGGTGCATTATGCATTG	TRMVHYALAS
	GCTTCTCTTCTGCATTCATTTGATTGGTCTATG	LLHSFDWSMP
	CCAGATGGGGAGAAATTGGACATGGCTGAAA	DGEKLDMAEK
	AATATGGTATTGTGCTAAGGAAGGCAGTTCCT	YGIVLRKAVPL
	CTTGTTGCTTTGCCTAAACCTCGTTTGTTGCAG	VALPKPRLLQS
	TCTGATCTCTACTATTAA	DLYY*
	ATGGATACCCTTCTTCCATTCCTATTCTCTGTT	MDTLLPFLFSV
	TCATTATCAGCGGCCATTATACTCCTAATTTCA	SLSAAIILLISFY
	TTTTATGGAGTGAATAGAAGAGGGGGAAAGC	GVNRRGGKLP
	TGCCGCCTGGGCCGCGAGGGCTGCCAATTTTG	PGPRGLPILGSL
	GGTAGTCTGCTCGACCTGGGTTCGAATCCCCA	LDLGSNPHQSL
	CCAGTCTCTGTATAATCTCTCCAAGCGCTATG	YNLSKRYGGL
	GCGGCCTCATGTATCTGAAAATAGGAACCACC	MYLKIGTTPAV
	CCCGCGGTTATAGCTTCCTCTCTCGAATCCGCA	IASSLESATAIL
	ACCGCCATATTGAAGACATTCGATTCCAATTT	KTFDSNFCNRP
	CTGTAACAGGCCGCAGGATGGCGGCGCCGCA	QDGGAAADIL
	GCAGATATACTCTTGTACAACAAAAACGACAT	LYNKNDISSS
	CTCCTCGTCCTCGAATTGGCCGATGCTGCGAA	NWPMLRKLCV
TcF3'H3	AGCTCTGCGTTGTCCACCTGTTGAGTCCCAAA	VHLLSPKCVEE
	TGTGTGGAGGAGTGGTCGGATTCCAGAGAAG	WSDSREEEMA
	AAGAAATGGCGCTGCTGCTCTCTTCCATCTTC	LLLSSIFKQRAT
	AAACAACGAGCTACTACCGCTGTGAACGTGGG	TAVNVGDYLN
	AGATTATCTTAACGTCTTCGCCTCTAATGTTAT	VFASNVISQM
	TTCTCAGATGATGTTGAAGATGAGAATTTTCG	MLKMRIFEDN
	AAGACAACAACGCAGAGGCGGCGCATTTCAG	NAEAAHFRDL
	AGATTTAATGGACGAATTCCTGTGCGCCACTG	MDEFLCATAR
	CTCGTTTCAGGATTGAGGATCATGTTCCTTTCT	FRIEDHVPFLG
	TGGGGAAGATTGGCTTCGGCGCTTCTCTGGTT	KIGFGASLVRE
	CGGGAAATGAAGAAGCTGCACGAGCGCATCG	MKKLHERIDEF
	ATGAATTCCTGGTGAGGAACATACGAGAGCAC	LVRNIREHSLIP
	AGTCTTATTCCCACCAACGGCAAGAAAAAAGA	TNGKKKDFVEI
	TTTTGTAGAAATTCTCGAAGCACTCAAATCCG	LEALKSDSHGQ

	ATTCTCACGGACAGCTTTCTAACTCGAATATC	LSNSNIKGILLN
	AAAGGCATTTTACTGAACATGTTTGCAGCAGG	MFAAGTDTAS
	AACCGACACGGCGTCGCGTACAGTGGAATGG	RTVEWAMSELI
	GCAATGTCCGAGCTGATCCGCCATCCTCACGT	RHPHVMKRVR
	AATGAAGAGGGTGAGAGAGGAAATCGATTCA	EEIDSEVGVEE
	GAGGTGGGAGTGGAAGAGAGAGTTAAAGAAA	RVKESDVPRLK
	GTGATGTTCCTCGGCTGAAATATTTAGCGGCA	YLAAVVAESL
	GTTGTGGCGGAGAGCTTGAGACTCCATCCTCC	RLHPPTPLMLP
	GACTCCGCTAATGCTTCCTCACGCATCTGGAC	HASGQASTVL
	AGGCCTCCACAGTGTTGGGACATTTCATCCCT	GHFIPRNTRVM
	CGAAACACCCGCGTCATGGTCAACGTGTGGGC	VNVWAIARDP
	TATTGCGAGAGATCCAAATTTATGGGAGAGGC	NLWERPLEFDP
	CGCTGGAATTCGATCCAGATCGATTTGTGGGT	DRFVGRSVNL
	AGATCAGTGAATCTTCACGGAACGGATTTTCG	HGTDFRIIPFGA
	AATAATACCGTTCGGCGCGGGGCGAAGGATGT	GRRMCPGYNL
	GCCCGGGCTATAATTTGGGCATGCGTATGATT	GMRMISFALAT
	AGTTTTGCACTTGCCACCTTCATTCATGCCTTC	FIHAFDWTLPA
	GATTGGACACTTCCTGCTCCTCAAAATCCCAG	PQNPRDLDMS
	AGATTTAGATATGTCTGAAAAATATGAAGTTT	EKYEVSIRRKV
	CTATTCGCAGGAAAGTTCCTCTCAAGCTTTTTG	PLKLFATPRLP
	CCACACCTCGCCTGCCTGCCCATCTCTATCTCT	AHLYLCNKS*
	GCAACAAGTCGTGA	
	ATGACTTCCGCTGTGGATTTTGTGAACTCTGTT	MTSAVDFVNS
	CCGTTGAGCGTAGGCCAGGCCCTGGGCCTGGC	VPLSVGQALGL
	GCTTCTGTTACTGGTCGGAATAATTTATATTGG	ALLLLVGIIYIG
	GAAGTCGAGACGAAGAAATCGTCTACCGCCC	KSRRRNRLPPG
	GGGCCAGCGCCATGGCCAGTGATCGGAAGCCT	PAPWPVIGSLP
	TCCGCTTCTGGGCACCATGCCTCACAGCTCGC	LLGTMPHSSLY
	TGTATCACCTGTCGAAGCAGTACGGGCCGCTC	HLSKQYGPLM
	ATGTATTTGAAGCTCGGCACCACCGACACCGT	YLKLGTTDTV
	GGTGGCCTCCTCGCCTAAAGTAGCCGAGGCTT	VASSPKVAEAF
	TTTTGAAGACAAATGATGGAAATTTCTCCAAC	LKTNDGNFSN
	AGGCCGGGCAACGCAGGCGCCAAATACATGG	RPGNAGAKYM
	CGTACGACTCCAACGACCTCGTCTGGGCGCCC	AYDSNDLVWA
	TACGGCAAGCGCTGGCGCATGCTGCGAAAGGT	PYGKRWRMLR
	CTGTAATATTCATTTGTTTGCCGGCAAGGCGCT	KVCNIHLFAGK
	GGACGACATGCAGCCGCTCAGGCAGGCGGAG	ALDDMQPLRQ
	GTAGGGTTGCTAGTCAGGTCTTTGCTGGAACA	AEVGLLVRSLL
TcF3'H4	CGGGCGGCGCGGTGAGGCGGTCAATTTGGGG	EHGRRGEAVN
	GAGATGTTGAATGTCTGCACCGCGAATGTGCT	LGEMLNVCTA
	CGGCCAGGTGATGTTCAGCAAGCGCGTGTTCG	NVLGQVMFSK
	AATCCCGGGGGGATTCGAAAGCGAGCGAGTTT	RVFESRGDSKA
	AGGGAGATGGTGATAGAGCTGATGGTGCTGG	SEFREMVIELM
	CTGGGGTGTTTAATATTGGCGACTTTGTGCCCT	VLAGVFNIGDF
	CCTTGGCTTGGCTCGATTTGCAGGGCGTTCAG	VPSLAWLDLQ
	GCCAAGATGAAGATTTTGCATGCTAAGTTTGA	GVQAKMKILH
	TGATTTCTTTGGGAGAATTATAGCGGAACACC	AKFDDFFGRII
	AAGAGGAATCTGCGAGGACGGGCAAAATTAA	AEHQEESARTG
	GAAGGATTTTCTTAGCGTTATTCTGGCGCTTAG	KIKKDFLSVIL
	AAATAATGCCGATGGAGAAGGCGGGCAGCTC	ALRNNADGEG
	ACTGATACCGACATGAAGGCGCTTTTGCTGGA	GQLTDTDMKA
	CCTTTTCACGGCAGGAACGGACACGTCCTCAT	LLLDLFTAGTD
	CGACGGTAGAATGGGCCATAACGGAGCTCATC	TSSSTVEWAIT
	CGGCACCCAGACATAATGGAAAAATGCCGGC	ELIRHPDIMEK

	AAGAACTGGACACGATGGTGGGAACCGAGCG	CRQELDTMVG
	GAAACTGGAAGAGAACGACCTCCAGAAGCTG	TERKLEENDLQ
	TCATATCTCCAAGCAGTGGTAAAGGAAACATT	KLSYLQAVVK
	CCGGCTGCACCCGTCGACGCCGCTCCTTCTGC	ETFRLHPSTPLL
	CACGAATGGCGGCGGAGGCGTGCACAATAGA	LPRMAAEACTI
	GGGGTACCACATTCCGAAGGGCGCGCGGCTG	EGYHIPKGARL
	ATGGTGAACGCGTGGGGCATACAGCGCGACC	MVNAWGIQRD
	CGGACGTGTGGGAGAGGCCGCTGGAGTTCGA	PDVWERPLEFD
	CCCGGAGCGCTTCGTGGGAAGCACTGTGGACG	PERFVGSTVDV
	TGCGGGGAACGGACTTTGAAGTAATCCCGTTC	RGTDFEVIPFG
	GGCGCGGGGAGGCGCATCTGTGCGGGCGTAA	AGRRICAGVS
	GCATGGGCATGCGCATGGTGCAGTTCATGCTC	MGMRMVQFM
	GCCACGCTCGTTTACTCCTTCGACTGGTCGCTT	LATLVYSFDW
	CCAGCGGGGCAGATGGCGGAGAAGCTGGACA	SLPAGQMAEK
	TGGCGGAGGCGTTTGGGCTCACCCTTCAGAAG	LDMAEAFGLT
	GCGGTGCCCCTCCTCGCCGTTCCGTCCCCTCGT	LQKAVPLLAVP
	TTGCCCCTTGCTCTCTACGACTGA	SPRLPLALYD*
	ATGGAGGTGGCTCTTTCCCCAGTCAGAGAAGT	MEVALSPVRE
	TTTACTGTGGGGGCTTTCATGGCTTTGTCTGTA	VLLWGLSWLC
	CATTGCATTTAGATATGTTCTGAGTAAGAGTA	LYIAFRYVLSK
	AAAAGCTTCCTCCTGGTCCTTCGGGATGGCCT	SKKLPPGPSGW
	GTTTTAGGCTGCTTACCATTGTTAGGCTCAATG	PVLGCLPLLGS
	CCTCATGTCGCTCTGACAAATTTGTCAAAGAA	MPHVALTNLS
	ATATGGCCCAATTCTTTATCTAAAATTGGGTA	KKYGPILYLKL
	CCTCTAATATGGTGGTGGCCAACACTCCTGCT	GTSNMVVANT
	GCTGCGAAGGCTTTTTTGAAGACTCTGGACAT	PAAAKAFLKTL
	TAATTTTTCCAACAGGCCTGGCAATGCTGGGG	DINFSNRPGNA
	CTACTTATTTGGCATACAACTCTCAGGACATG	GATYLAYNSQ
	GTGTGGGCACCCTACGGTGGAAGATGGAAGA	DMVWAPYGG
	TGCTCAGAAAGGTGTGCAATCTCCACATGCTA	RWKMLRKVC
	GGAGGCAAGGCCTTAGATGACTGGCAGCCTGC	NLHMLGGKAL
	GAGGGAAGCCGAGATGGGTCACATGTTGAGG	DDWQPAREAE
	TCAATCCTCGCTCGTTCCCGCAGTGGGCAGAC	MGHMLRSILA
	TGTGAACATCCCTGAACTGCTCAACCTGTTGG	RSRSGQTVNIP
	CAGCCAACGTTTTTGGGCAGATTATCCTGAGC	ELLNLLAANVF
TcF3'5'H	AAGCGAGTTTTTGAGAGCGAGGGGGAGGATG	GQIILSKRVFES
	CCAACGAGTTCAAGGAGATGGTAGTCGAGCTC	EGEDANEFKE
	ATGACTACCGCGGGGTATTTTAACATAGGCGA	MVVELMTTAG
	TCATATCCCCTCCATTGCATGGATGGATTTGCA	YFNIGDHIPSIA
	GGGAATACAGCGGGGTATGAAGAAACTACAT	WMDLQGIQRG
	AAGAGATTTGACGAAATGCTAACCAGGATGAT	MKKLHKRFDE
	TGCAGAACACCAGGCGGCGGCCAAGAAGCGA	MLTRMIAEHQ
	GCTGCTCCAGACTTCTTGGACATTGTAATGTCC	AAAKKRAAPD
	CAACGGGATAACTGTGATGGCCAGGGAGGAA	FLDIVMSQRDN
	GGTTGTCTGACGTTCACATCAAGAGTCTACTTT	CDGQGGRLSD
	TGAATTTGTTCACAGCGGGGACCGACACTTCA	VHIKSLLLNLF
	AGCAGTGTTATAGAATGGACGCTTGCAGAGTT	TAGTDTSSSVI
	AATTCAAAACCCTAAGCTTCTTAAGCAGGCAC	EWTLAELIQNP
	ATTTAGAGATGGACAAAGTGGTTGGTCGTGAT	KLLKQAHLEM
	AGGCGACTTAAAGAGTCAGATATTCCAAAGCT	DKVVGRDRRL
	CCCCTACCTGATAGCCATTTGCAAGGAAGGCT	KESDIPKLPYLI
	TCCGGAAGCATCCCTCCACTCCTCTCAGCCTA	AICKEGFRKHP
	CCACGAGTCTCTTCTGAGCCTTGTCAAGTAGG	STPLSLPRVSSE
	TGGCTACTATGTGCCCAAGGGAACTCGTCTCA	PCQVGGYYVP

	TGGTCAACATTTGGGGCATCGGTAGGGATCCT	KGTRLMVNIW
	GAAATATGGGACAAACCCCTTGAATTCAATCC	GIGRDPEIWDK
	AAACAGGTTTGTGGGATCCAAAATTGATCCAC	PLEFNPNRFVG
	GTGGAAATGACTTTCAACTCATACCTTTTGGT	SKIDPRGNDFQ
	GCGGGAAGGCGTATTTGTGCTGGTACTAGGAT	LIPFGAGRRICA
	GGGGATTACCATGGTGGAATACAATTTGGGGT	GTRMGITMVE
	CGTTGATCCATGCTTTTAATTGGGAAATGCCA	YNLGSLIHAFN
	CCAGGTAAAGAGAGCTTGAACATGGATGAGT	WEMPPGKESL
	CTTTTGGACTTGCGCTACAAAAGAAAGAGCCC	NMDESFGLAL
	CTTGTGGCCAAGGCTACCCCTCGCCTTGCCCTT	QKKEPLVAKA
	CACCTATACTAG	TPRLALHLY*
	ATGGGGTTGACAGAAGCCGTGTGCGTGACCGG	MGLTEAVCVT
	AGCTAGCGGGTTTATCGGTTCATGGGTCGTTC	GASGFIGSWVV
	GTTTGCTCTTGGAACGGGGTTATGCCGTGCAT	RLLLERGYAV
	GCCACCGTTCAAAATCTCGAAAATTGGAAGGA	HATVQNLENW
	AACTAAGCATTTGGAGGCGATGGAAGGAGCC	KETKHLEAME
	AAAGAAAGGCTGAAGTTATTTCAAATGGATTT	GAKERLKLFQ
	ACTGAACTACGAGTCAATTGAGGCGGCCATTA	MDLLNYESIEA
	ATGGATGCGCTGGTGTTTTCCATTTGGCAATG	AINGCAGVFHL
	CCCAATACTATTGAGGCCGTTCAGGATCCACA	AMPNTIEAVQ
	AAAGCAACTGATGGACCCTGGCATCAAGGGC	DPQKQLMDPGI
	ACTCTGAACGTACTGGAAGCAGCCCTTAAATT	KGTLNVLEAA
	CCAAGTGAAGCGGGTGGTGCTCACCTCTTCTG	LKFQVKRVVL
	TTTCGGCCTTCGTTCCCAACCCCAAATGGCCTG	TSSVSAFVPNP
	CTGAGACCCCCTTGGACGAATCCAGCTGGACC	KWPAETPLDES
	GACCTCGAATACTGCAAACAAAATGGGATTTG	SWTDLEYCKQ
TcDFR1	GTACCCTGTGGCAAAAACACTGGCGGAAAAG	NGIWYPVAKT
	GCAGCATGGGACTTCAGCAAAGAGAGAGGGT	LAEKAAWDFS
	TGGATATAGTTGCTATTAACCCTGGGACTGCA	KERGLDIVAIN
	TTGGGCACAATTCTGCCTCCTGATTTCAATGCC	PGTALGTILPP
	AGCTTGGCTATGATCGTCCGCCTGGTCGATGG	DFNASLAMIVR
	TAACAAAGAGGAGTATCAAAACTTTTACATGG	LVDGNKEEYQ
	GCTGTGTTCATGTGAGGGATATCGCAAAATCT	NFYMGCVHVR
	CAAATAAAATTATATGAAACACCCTCAGCATC	DIAKSQIKLYE
	GGGACGACATCTCTGTGTCGAATCCATCACCC	TPSASGRHLCV
	ATTGGAGCGACTTTGCAGAGTTGACAGCAAAA	ESITHWSDFAE
	CTATATCCAGAATATAATGTGCCCAAGTTCAC	LTAKLYPEYN
	AAATATTACTCAACCCGGGTTGGTACGTGTGA	VPKFTNITQPG
	AGAATGCACCCAAGAAACTGATCGATCTTGGG	LVRVKNAPKK
	GTAGAATTTGCTCCGATGGAGCAAATCATTAA	LIDLGVEFAPM
	AGATAGCGTTTCATCGTTGAAGGAGAAAGGTT	EQIIKDSVSSLK
	TTCTCGATTAA	EKGFLD*
	ATGACTGTTTTTCCTCATTTGGATGAAGGAAA	MTVFPHLDEG
	TGGCGGGGGAAAAGTTGTGTGTGTAATGGATG	NGGGKVVCV
	CCTCCAGTTATGTTGGCCTCTGGATTGTTCAGG	MDASSYVGLW
	GTCTTCTACACAGGGGTTACACTGTACATGCC	IVQGLLHRGYT
	ACAGTCCAGAATGGAGGTGAAGCTGAATCTTT	VHATVQNGGE
TcDFR2	AATGAAATTGAATGGGGAGCGGTTGAAGATCT	AESLMKLNGE
	TCTATGCAGATATGTTGGATTATCACAGCATT	RLKIFYADMLD
	GTGGATGCACTGAATGGATGTTGTGGCCTTTT	YHSIVDALNGC
	CTACACGTTTGATCCCCCTCAATATGATGAGG	CGLFYTFDPPQ
	TGATGGCTGAAGTTGAGGTGAGGGCAGCCCAC	YDEVMAEVEV
	AATATCTTGGAAGCCTGTGCTCATACTGAAAC	RAAHNILEACA
	ACTCCAAAAAGTGGTATTCACATCTTCGGTGG	HTETLQKVVFT

	CTGCGGTAATTTGGAGAGATGACAGAAATTCA	SSVAAVIWRD
	ATTGCTGACCTCCATGAGAGACATTGGAGTGA	DRNSIADLHER
	TGTAAACCTTTGCAGAAGATTGAAGCTGTGGT	HWSDVNLCRR
	ATGCATTGGGCAAGACACTGTCAGAGAAGAC	LKLWYALGKT
	AGCATGGGCCCTGGCAATGGACAGAGGAGTG	LSEKTAWALA
	AATATGGTGACAATCAATACAGGTCTAGTTGT	MDRGVNMVTI
	AGGGCCTGGCTCTGCATACAAAACCTCAGGAT	NTGLVVGPGS
	CCACCATTGCATATCTTAAAGGTGCTGCACAG	AYKTSGSTIAY
	ATGTACGAGAATGGATTGTTAGCAAGTGCAGA	LKGAAQMYEN
	TGTAAGGTTTGTAGCAGAGGCCCACATCTCTG	GLLASADVRF
	CATTTGAGGACCCCTCTGCTTTTGGGAGATAC	VAEAHISAFED
	ATCTGTTTCAACCAAATTGTCAACAATCCCCT	PSAFGRYICFN
	GAATGCTGACAGTCTTGCTGAGAGTTTGAGAC	QIVNNPLNADS
	CGCTCATACCATTTCCTGACAGATGTGAAGAT	LAESLRPLIPFP
	TCAAATGTCTACCAACAACGACTGAACAACAA	DRCEDSNVYQ
	GAAGTTGAGTGAACTCATGAGTGGATACACAA	QRLNNKKLSEL
	GGCAATGTAGGATGGAATAA	MSGYTRQCRM
		E*
	ATGGGTTTTGGGGACGAGACTTCTTCCGTAGT	MGFGDETSSV
	CATGTCAGAAGCCGCCACAAAGATACCAACCC	VMSEAATKIPT
	AGAAAGAAGAGAGAGGAACAGTGTGTGTCAC	QKEERGTVCV
	TGGGGCTGCTGGATTCATAGGATCATGGCTTA	TGAAGFIGSWL
	TTATGCGCTTACTTGAGAGGGGATACACTGTG	IMRLLERGYTV
	AGAGCAACTGTTCGAGACCCAGGAAACCCAG	RATVRDPGNP
	TGAAGACCAAGCATTTGTTGGATCTCCCAAAG	VKTKHLLDLP
	GCCAGTGAAAGATTGACACTTTGGAAAGCAG	KASERLTLWK
	ATCTGGATGATGAAGGAAGCTTTGATGCTGCC	ADLDDEGSFD
	TTTGATGGTTGTGAAGGTGTTTTCCATGTTGCC	AAFDGCEGVF
	ACTGCCATGGACTTTGACTCTAAGGACCCGGA	HVATAMDFDS
	GAATGAGATAATTAAGCCATGTGTGGCTGGGG	KDPENEIIKPCV
	TGTTGAATGTTATGAGATCGTGTGCCAAGGCC	AGVLNVMRSC
	AAGTCTGTGAAGAGAGTTATTTTTACCTCATCT	AKAKSVKRVIF
	GCTGGGACAGTGAACTTCACTGATGATTGTCA	TSSAGTVNFTD
	AACACCTGGCAAAATTTTTGATGAAGAATGCT	DCQTPGKIFDE
TcDFR3	GGACCAATGTGGATCTTTGCAGGAGTTCAAAA	ECWTNVDLCR
	ATGACTGGTTGGATGTACTTTGTGTCAAAGAC	SSKMTGWMYF
	TTTGGCAGAAAGAGCTGCCTGGGAGTTTGCAG	VSKTLAERAA
	AGCAGAATAAGGTTGATCTGATTTCAGTGATA	WEFAEQNKVD
	CCAACATTGGTGGTTGGGCCATTCATCATGCA	LISVIPTLVVGP
	GACTATGCCTCCCAGCTTAATCACTGCTTTAGC	FIMQTMPPSLIT
	ACTCTTAACAAAAAATGAACCCCACTACATGA	ALALLTKNEPH
	TATTGAGACAAGTGCAGCTGGTTCACTTGGAT	YMILRQVQLV
	GACCTCTGCATGTCACTCATTTTCCTGTATGAA	HLDDLCMSLIF
	CATCCTGAAGCAAAAGGAAGATACATCTCTTC	LYEHPEAKGR
	TTCTCATGATACTACCATTGTTCAAGTGGCAA	YISSSHDTTIVQ
	AGATGTTAGCTAAGAAATATCCACAGTACAAT	VAKMLAKKYP
	GTTCCAACTAAATTCAAGGATGCAGATGAGTC	QYNVPTKFKD
	TTTGCAGCCTGTGGCATTTTCAAACAAGAAAC	ADESLQPVAFS
	TTGTTGACCTGGGATTTAAATTCAAGTACACA	NKKLVDLGFK
	CCAGAAGACATGTTTGATGGGGCCATCCAGTG	FKYTPEDMFD
	TTGTCTTGAGAAAGGATTGCTGAAGTAG	GAIQCCLEKGL
		LK*
TcDFR4	ATGGCGGAACAGACAAAGAAGAGAGTGTGCG	MAEQTKKRVC
	TGACAGGTGCCTCGGGATACATTGGATCCTGG	VTGASGYIGS

	CTTGTCAAAAATCTCTTACAGAGGGGTTATGC	WLVKNLLQRG
	AGTTAATGCAACCCTAACAGATCCAGGAAATG	YAVNATLTDP
	AGATAAAATCTGGAGCTTTAATGGACCTGCCT	GNEIKSGALM
	GGGGCAAAAGATTGTCTTAAGCTCTTTAAAGC	DLPGAKDCLK
	AGATTTGTGTGATGAAGGAAGCTTTGACTCTG	LFKADLCDEGS
	CCATACAGGGTTGTCAAGGTGTTTTTCATGTTG	FDSAIQGCQGV
	CAGGTCCCATGGATTTTGCAAAAAAATCTAAA	FHVAGPMDFA
	GAAGATTTTGTTGAAACTGCTGTAAATGGAGT	KKSKEDFVETA
	AGTGAATGTTATGGAGGCTTGCACAAGGGCCA	VNGVVNVMEA
	AATCTGTTAGCCGAGTGGTTTTCACATCTTCTG	CTRAKSVSRV
	TCGTAGCAGCTTGTCCAATGAATGACAAGGGG	VFTSSVVAACP
	GAAGTAGAACAAACATGCGTTGATGAAAGGT	MNDKGEVEQT
	GTTGGAGCCCTCTAAATTTTCTTGAATCTCAGA	CVDERCWSPL
	CATCTAAACTGGCCTGGTATGCGACTGCCAAG	NFLESQTSKLA
	ACATTATCAGAAAAAGAGGCCCTTAAATACGG	WYATAKTLSE
	TGGCGATAACAAATTGGAGGTTGTGACTGTCC	KEALKYGGDN
	TACCAGCTGTGGTACTTGGCCCTTGGTTTACA	KLEVVTVLPA
	GCCACACCTCCTTTATCAACACTCCAAACAAT	VVLGPWFTAT
	TTTAGCCCTGATTGGAGGAAATGATGAGTTTT	PPLSTLQTILAL
	ATGAATATTTAAAGCTCATGGAATTTTTGTTG	IGGNDEFYEYL
	GGATCCATACCAATTGTTCATATTGAGGACGT	KLMEFLLGSIPI
	GTGCAATGCCCACATATTGTTGATGGAACATC	VHIEDVCNAHI
	CAGATGCCCAAAACCGTCATTTTTGTGCATGT	LLMEHPDAQN
	GGTTCTCGGAGTCTCAAGTGTCTCAAAGATTA	RHFCACGSRSL
	TCTGGCTAAACATCGTGTGCAATCCCAGGCGA	KCLKDYLAKH
	CTGTGAAGTTAGATGAGGAGGAAGGTCGAGC	RVQSQATVKL
	TCATGCATATCTCCCAACATCTTCAAGGAAAT	DEEEGRAHAY
	TGCTGGATATGGGCTTCACTTACAAGTATAGT	LPTSSRKLLDM
	TTAGAAGAGAGTTTTGACGAGGGAATAGAGT	GFTYKYSLEES
	GTGCCATGAACAATGGAATCTTGAAGTTGTAA	FDEGIECAMNN
		GILKL*
	ATGCAGCAGTCGAGGTTGGAAACCGTGTGCGT	MQQSRLETVC
	GACAGGGGCAGGAGGGTTTTTGGCCTCATGGC	VTGAGGFLAS
	TTGTCAAATTACTTCTCGAACGCGGTTATAAC	WLVKLLLERG
	GTCCGCGGCACCGTCCGAGACCCCGACGATGC	YNVRGTVRDP
	AAAGAATGCACATTTGAAAGACCTAGAAGGG	DDAKNAHLKD
	GCAAAGGATGGGCTAAAACTCCTAAAGGCAG	LEGAKDGLKL
	ATTTGTTGGACTATGGCTCACTTTCTGCAGCAA	LKADLLDYGS
	TTGATGGGTGCACTGGAGTTTTCCACACAGCA	LSAAIDGCTGV
	TGTCCAGTTCCTTCTCACAGAGTGTCTGACCCA	FHTACPVPSHR
	GAGGCCGAGGTTCTTAATCCTGCTGTAAATGG	VSDPEAEVLNP
	AACTCTGAATGTATTGAAGGCATGTTCAGTGG	AVNGTLNVLK
TcDFR5	CTAAGGTGAAGCGTGTAATTATGACATCGTCT	ACSVAKVKRVI
	GTAGCTGCTATTTTTCTAAACCCAAATCGGCC	MTSSVAAIFLN
	AATGGATACACTTCTAGATGAGAGCTGCTGGT	PNRPMDTLLDE
	CAGATCCAGAGTACTGTAAGGCAGCTCAGAAT	SCWSDPEYCK
	TGGTATTTCCTGTCAAAAACAGTCTCCGAACA	AAQNWYFLSK
	GGGTGCCTGGAACTTTTCAAGGGAGAATGGCC	TVSEQGAWNF
	TCGACCTTATCACCATATGTGCATCCTGGATTT	SRENGLDLITIC
	TAGGACCTATGTTACAGACAACTATGAATGCT	ASWILGPMLQT
	AGTTGTCTATCTCTTTTCAAGCTTTTGACTGGT	TMNASCLSLFK
	GAATATGAGAGGCGTGATAACAAAGTAATCC	LLTGEYERRDN
	ATATGATAGATGTCAGAGATTGTGCGAAATCA	KVIHMIDVRDC
	CATGTACTTGCTTATGAAGCTCTTTCTGCAGCA	AKSHVLAYEA

	GGACGATATCTATGCACAGCTCATAAATTTAA	LSAAGRYLCT
	AACCACGGAACTGATTGACATCCTTCAAAGGC	AHKFKTTELIDI
	TTTACCCACAGTATACCTATCCCAAAGATTTTG	LQRLYPQYTYP
	TGGATGTAGAGTCAAATGGATCAGGGATTGAG	KDFVDVESNG
	CGATTATCAAATAGTAAACTGCAAGAGTTGGG	SGIERLSNSKL
	TTTGGAGTTCACGAAGTTGGAAGAGACACTAA	QELGLEFTKLE
	TTGATACTGTCAAATGTTTCCAGAAGAGGAAT	ETLIDTVKCFQ
	ACTTTGAAGTGA	KRNTLK*
	ATGGTCAAAAATCTGCTGGATAGAGGTTATAC	MVKNLLDRGY
	AGTTCACGCTACTGTAAGAGATCCTGAAAACC	TVHATVRDPE
	CTGCTAAGACTAGCCATTTGCTTTCCCTTCCGG	NPAKTSHLLSL
	GAGCCAAAGAGAGGCTCCATCTTTTCAAAGCA	PGAKERLHLFK
	GACTTATCACACCAGGGCAGCTTCGATTCCGC	ADLSHQGSFDS
	CATTGAAGGCTGTGACTTCCTTATTAACTTGGC	AIEGCDFLINL
	CACATCTATGGAATTCGATTCTAATGACCCTG	ATSMEFDSNDP
	AGACAGGATTTATTCGATCTACCATTGATGGA	ETGFIRSTIDGT
	ACTGTTGACATTTTGAGAGCTTGTAAGAAAGC	VDILRACKKA
	AAAAACTGTGAGACGGGTGATCCATACATCCT	KTVRRVIHTSSI
	CTATAACGGCAGCTTCCCCGCTGGATGAAAAT	TAASPLDENGN
	GGAAATTTCAAGGAGTGTCTGGACGAATCTTG	FKECLDESCWT
	CTGGACTCCTGTGGATTATATTAGGGCTAAAA	PVDYIRAKKPK
	AGCCCAAATATTGGATGTATTATGTGACAAAG	YWMYYVTKTL
	ACATTAGCTGAGCAAGCAGCTCTGCAATGCAG	AEQAALQCSA
TcDFR6	CGCAGATGATGAAATTGAAGTGGTAACAGTGT	DDEIEVVTVCV
	GTGTAGCTCTAGTGGGTGGCCCATCATTCACC	ALVGGPSFTPT
	CCGACACTTCCCTCAAGCATTTCACAGATCTT	LPSSISQILTPIT
	AACTCCAATCACAGGTAACAGAGAACTTTGTG	GNRELCETVNE
	AAACCGTGAACGAACTTCAATCTTTGACGGGC	LQSLTGSIPMV
	TCCATTCCAATGGTCCACATAGAAGATGTATG	HIEDVCHTHIF
	CCACACCCATATCTTCCTCATGCAGCACCCCTT	LMQHPFAVGR
	TGCAGTGGGTCGATACCTGTGCTGCTCAGATG	YLCCSDALTIA
	CGCTCACCATTGCTGACATGGCAGATTTCTTTA	DMADFFSKRH
	GTAAACGCCATCAACCAGTCCACATTGCCTTT	QPVHIAFKNM
	AAAAATATGGTGAAAGATGAGGTGAAGGGCT	VKDEVKGFVP
	TTGTACCGGTCTCTTCAAAGAAATTAACTGAA	VSSKKLTELGF
	CTGGGTTTCTCATACAAGTATGGGATGGAAGA	SYKYGMEEIV
	AATTGTGGATCATGGTATTCAATGTGCAAAGA	DHGIQCAKKM
	AGATGCGGGTCTTATCACAGGAATGCTAA	RVLSQEC*
	ATGGTCAAAAATCTGCTGGATAGAGGTTATAC	MVKNLLDRGY
	AGTTCACGCTACTGTAAGAGATCCTGAAAACC	TVHATVRDPE
	CTGCTAAGACTAGCCATTTGCTTTCCCTTCCGG	NPAKTSHLLSL
	GAGCCAAAGAGAGGCTCCATCTTTTCAAAGCG	PGAKERLHLFK
	GATATCTATGAACAAGGTAGCTTTGATTCAGC	ADIYEQGSFDS
	TATCCAAGGCTGCGACTTCCTCATCAATTTGG	AIQGCDFLINL
	CCACCGGTATGGATCCCCAGAGAGGGTTTGTC	ATGMDPQRGF
TcDFR7	GACCCTGTCGTTGAGGGGACGCTTGACATTCT	VDPVVEGTLDI
	AAGAGCCTGCAAGAAATCAAAAAGCGTGAAA	LRACKKSKSV
	CGGGTGGTCCATATTTCCAGCATAGGCGCAGC	KRVVHISSIGA
	TTTCCCCCTGAATGAAGAGGGAAAACATAAGG	AFPLNEEGKHK
	ACCTCCTTGACGAATCCTGCTGGACTCCTGTTG	DLLDESCWTP
	ATTATATGACAAGAAAAAGCCCTGATATGGGG	VDYMTRKSPD
	ATGTATTGTGTGTCCAAAACCCTAGCGGAGCA	MGMYCVSKTL
	AGCGGCGCTGCGATACGGGGAGGAGGAGGGA	AEQAALRYGE
	ATTGAAGTGGTTACACTGTTGGTGGCAATGGT	EEGIEVVTLLV

	GGGCGGATTTTCCCTCGCCCCTGGATTTCCCA	AMVGGFSLAP
	GCAGCCTTTCAATTATCTTAGCACCAGTCACA	GFPSSLSIILAP
	GGCAACAGAGCATGTTATGAAACCTTGGTTTC	VTGNRACYET
	ATCCCAATTTTTGATGGGCTCCATGCTATTGGT	LVSSQFLMGS
	CCATTTGGAAGATGTATGCAACGCCCATATTT	MLLVHLEDVC
	TCTTGATGGAGCACCCATCTGCAGCGGGTCGA	NAHIFLMEHPS
	TATGTGTGCTGTTCAGACTCTGTGACCATGGCT	AAGRYVCCSD
	GACATCGCAGATTTCTTTCGCAGACGCTATCC	SVTMADIADFF
	AGAAACCCCCCTGGTGTTTGGTGATAAGGAGG	RRRYPETPLVF
	AAGATCGGCTGGAGAAACTTGTGCCATTTTCT	GDKEEDRLEK
	TCCAAGAAATTAACCGATCTGGGATTTTCGTA	LVPFSSKKLTD
	CAAGTATGGTATGGAAGAAATATACAGCGAC	LGFSYKYGME
	AGCTTTGAATGTGCTAAGCAGATGAAAGTATT	EIYSDSFECAK
	GTGA	QMKVL*
	ATGGGTGTGGAAAGAGCACATTGGGAGAAGG	MGVERAHWE
	TGTGTGTGACTGCAGCAGGAGGATTCATCGGC	KVCVTAAGGFI
	TCCTGCCTTGTGAAAAATCTGTTAGGAAAGGG	GSCLVKNLLG
	TTATATTGTTCACGCCACCTGCAGAGATCCGG	KGYIVHATCR
	CAAATCTATCTAAAACCAGCCATCTGCTATCT	DPANLSKTSHL
	TTACCGGGAGCCAAAGAGAGGCTTCACCTCTT	LSLPGAKERLH
	CACAGCGGACTTATATGAGCCAGGCAGCTTCG	LFTADLYEPGS
	ATTCTGCCATTGAAGGCTGTGATTTCGTCATTA	FDSAIEGCDFVI
	ACTTGGCTACATCACTTGATTATTCAAGGGAT	NLATSLDYSRD
	CCAGAGACAGGTTTTATCCAGTCCACAATTGA	PETGFIQSTIDG
	TGGCACCCTGGACATTTTAAGAGCATGCAAGA	TLDILRACKKA
	AGGCTAAAACCGTTAGGCGGGTAGTCCATACT	KTVRRVVHTSS
	TCTTCGATTACAGCAGCCATGCCCCTTAACAA	ITAAMPLNKN
TcDFR8	AAATGGAGAATTCAAGGAGTGCCTTGACAAAT	GEFKECLDKSC
	CATGCTGGACTCCTGTTCATTATATGAGAGCC	WTPVHYMRAK
	AAAAAGCCTTTATACTGGATGTATTATGTGGC	KPLYWMYYV
	GAAGACACTAGCGGAAGAAGCAGCTCTTCAA	AKTLAEEAAL
	TACGGGAAAGATGAGGGGCTTGAAGTGGTAA	QYGKDEGLEV
	CGGTTGCAGCAGGGTTAGTGGGTGGGCCGTCA	VTVAAGLVGG
	CTAGCACCAACGTTTGAATCTTGCAGCGCATT	PSLAPTFESCSA
	TATAACGTTAGCGCCTATCACAGATCTTTTTCG	FITLAPITDLFR
	TAAACGGTATCCAGACATGCACGTAACAGCCT	KRYPDMHVTA
	TCGATTATAAGGAAGATGATCAGCCGCCATTG	FDYKEDDQPPL
	CCAATATTTTCAAAGAAATTGGCAGAACTGGG	PIFSKKLAELGF
	ATTTTTATACGAATATGTCATGGAACAAATCG	LYEYVMEQIV
	TGGATGACAGCGTTGAATGTGCAAAGCAGATG	DDSVECAKQM
	AATCTTATATAAATAG	NLI*
	ATGGCCGCCACGGCAATTAACGGCAAAGCCG	MAATAINGKA
	CTGCAGCAATAGACGGCAGTAGCCCTGCAACT	AAAIDGSSPAT
	GCGATAGTGTGCGTAACGGGAGCGGCCGGTTT	AIVCVTGAAGF
	CATGGGCTCTTGGCTGGTGAAGCGCTTGCTGG	MGSWLVKRLL
	AAAAGGGCTACACAGTCCACGGCACAGTGCG	EKGYTVHGTV
	CGACCCAGACAATAAGGCAAAGGTATCCCATC	RDPDNKAKVS
TcANR	TGCTGAGCCTCCCCGGAGCCGAAGAACGGCTC	HLLSLPGAEER
	AAGCTTTTCCGGGCAGAGTTATCGGAGGAGGG	LKLFRAELSEE
	CAGCTTCGATGCCGCTGTTGCTGGTTGTGACG	GSFDAAVAGC
	GAGTTTTTCATGTCGCCACTCCCACCGAATTCG	DGVFHVATPTE
	CCCCAAAAGATCCCCAGAACGATTTAATCAAG	FAPKDPQNDLI
	CCTGCAATCGAAGGGACTCTGAACGCTCTGAA	KPAIEGTLNAL
	GGCTTGTACCAAGGCCAAAACCGTGAAGCGC	KACTKAKTVK

	ATAGTGGTCACATCCTCGGCCGCCACGGTTTC	RIVVTSSAATV
	CATTAACGAGTCCCCAGAGCAGAACCAGTATA	SINESPEQNQYI
	TCGACGAGTCTTGTTGGACAGATGTCAATTTT	DESCWTDVNF
	CTCCAGACCAAAACGCCCCCGGCTTGGGCATA	LQTKTPPAWA
	TCCAGTATCTAAGACACTTGCTGAGCAAGCTG	YPVSKTLAEQA
	CCTGGGAATATGCTAAGAAGCATGGTTTGGAC	AWEYAKKHGL
	ATGGTCACCATTATTCCCGTGCTAGTTGTTGGA	DMVTIIPVLVV
	CCTGCAATTACACCCATGGTGCCCTCTAGCAT	GPAITPMVPSSI
	TCAGCTTGCACTCTCTCCACTAACAGGAAATC	QLALSPLTGNP
	CACAGTTCTTTGGAGGCTTGAAGGGCATGCAA	QFFGGLKGMQI
	ATTGTATCAGGTTCCATTTCTTTAGTACATGTG	VSGSISLVHVE
	GAGGACGTCTGTAGTGCTCAAATATTCTTGAT	DVCSAQIFLME
	GGAGAATCTTAAAGCTCAAGGCAGGCATATTT	NLKAQGRHICC
	GTTGTCCCATTAACACATCTGTTCCACAACTTG	PINTSVPQLVE
	TAGAATACTTGGCTAAGCGCTATCCACATTAC	YLAKRYPHYN
	AACGTGCCTACTAAGATTGAGGATGTGCCTCC	VPTKIEDVPPTP
	CACCCCGAAAGTGAACATTTCATCCAAGAAAT	KVNISSKKLVK
	TAGTTAAGAGTGGCTTCACTTTTCAATATGGG	SGFTFQYGINDI
	ATAAATGATATTTACGACCATGCTGTTGAATA	YDHAVEYFKT
	CTTCAAGACTAAGGGCTTGCTCGCTTAG	KGLLA*
	ATGGATGGCAATTCCGAGAATGGTAATATGCA	MDGNSENGNM
	GAGAAAATTAAGCAGGGTGTTGATCATTGGAG	QRKLSRVLIIG
	CGACTGGTTACATGGGTGCTTTTGTTGCCAGG	ATGYMGAFVA
	GCGAGCTCAGATTCTGGTCACCCAACATTTGC	RASSDSGHPTF
	TCTTGTTCAGCCTTCAACTCTACAGGATCCTGG	ALVQPSTLQDP
	CAAGGCTGAATCCATAGACAATTTAAGGTCTT	GKAESIDNLRS
	CTGGGATTCACATCATACCTGGATGTCTGGAG	SGIHIIPGCLED
	GACCATGAATTGCTTGTAAAAGCAATTGCAGA	HELLVKAIAEV
	GGTAGATGTTGTGATTTCTGTGGTGGGTGGAG	DVVISVVGGV
	TCCAAACCATGGAGCAGTTGAAGATAATCGAT	QTMEQLKIIDA
	GCAATCAAACAAGTGGGCACTGTCAAAAGGTT	IKQVGTVKRFL
	TTTGCCATCGGAATTTGGGCACGATGTTGATA	PSEFGHDVDRA
	GAGCTGACCCAATTGAACCCGCTCAAAGTTTT	DPIEPAQSFYEE
	TATGAAGAGAAGAGAAAAATAAGAAGAGCCA	KRKIRRAIEEA
	TTGAGGAAGCCAAGATTGATTACACATATATT	KIDYTYICCNSI
TcLAR1	TGTTGTAACTCTATAGCTGGATGGCCGTATCA	AGWPYHYDTH
	TTATGACACCCATCCTTCTAAGCTCCCCCCTCC	PSKLPPPRDKM
	CAGAGACAAGATGGGCATCTATGGAGATGGC	GIYGDGNIKAY
	AATATTAAAGCATATTTTATGACAGGAAGAGA	FMTGRDIGVYT
	TATAGGGGTTTACACAATTAGGGCCATAGAAG	IRAIEDPRTLNK
	ATCCCAGGACTCTGAATAAGAAGCTTCATTTT	KLHFRPPGNFV
	AGGCCACCAGGTAATTTTGTGACAATGAACGA	TMNELADIWE
	ATTGGCAGACATATGGGAGAAGCATTTGGGA	KHLGKKIPRGV
	AAGAAAATACCCAGGGGTGTTATTAGTGAAG	ISEEDLLNCAR
	AAGATTTATTGAATTGCGCAAGAGCGAATGTG	ANVMPASIVA
	ATGCCAGCAAGCATAGTGGCAGCCATAACTCA	AITHDIFIKGCQ
	TGACATATTTATCAAAGGATGTCAATATAATT	YNFLIEGDEDE
	TTTTAATTGAAGGTGATGAGGATGAGGAGGCA	EACQLYPEITY
	TGCCAACTCTATCCAGAAATCACCTACACCAA	TNLEDYFRSLS
	TTTGGAGGATTACTTTCGAAGCCTTTCTTCAGA	SDL*
	TTTGTAA	
	ATGGCGTGCGGTCCTAATGTTGCGCACCAGTT	MACGPNVAHQ
TcLAR2	CAATGTTTCGTGCCTGAAGCCGCCAAAAGACG	FNVSCLKPPKD
	GCGCCTTGACTATCAACAACATTCTTACCAGC	GALTINNILTSN

AATGGCGGCTGCAACAACAATGCTGAGAATA	GGCNNNAENT
CGGCGGCTTCGCAGAATTTTGGTATTTGCAAG	AASQNFGICKG
GGGCCGCAAGTTAACGTGAGCTGCAAGGAGT	PQVNVSCKESC
CCTGTAGCAACGGTCATGTTAACGTGGGCAAG	SNGHVNVGKE
GAGTCCTGCAACGGTCATGTTAGCTTTAGTAA	SCNGHVSFSNG
CGGTCATGGTAGTGCTCTGAACAAGGAGAATG	HGSALNKEND
ATAACTTGGATGTGTTGACCAGTAAGGTTCTC	NLDVLTSKVLII
ATTATAGGAGCCACTGGTTTTATTGGACACTTT	GATGFIGHFVA
GTTGCACAGGCCAGTGTCAAGTCTGGGCGCCA	QASVKSGRQT
AACTTATGCTTTTGTGAGGCCTGGCACTTCCA	YAFVRPGTSKQ
AGCAGAAGGTTGTGCAGGCCTTGAGCGACTCC	KVVQALSDSG
GGAGTTCATATTCTTTATGGGTGTTTGGAGGA	VHILYGCLEDY
CTACAATTCTTTAGTGAGAGCCATAAGGCAGG	NSLVRAIRQVD
TGGACATTGTTATTTCTACTGTGGGGGGACCC	IVISTVGGPQIL
CAAATTCTCAGCCAGCTTAGGATTATAGACGC	SQLRIIDAIKEV
CATTAAGGAAGTGGGTGCTACAGTTAAGAGGT	GATVKRFLPSE
TTCTTCCTTCTGAATTTGGGCACGATGTAGACC	FGHDVDQADP
AGGCAGATCCGGAGGAGCCTGCACTGACATTT	EEPALTFYIEK
TACATTGAAAAGAGAAAGATCCGTCGCGCTGT	RKIRRAVEEA
GGAAGAAGCCATGATTCCTTACACATACATAT	MIPYTYICCNSI
GCTGCAACTCCATAGCGGGCTGGCCTTATTAT	AGWPYYYHTH
TACCATACACATCCCACAGAGCTGCCTCCTCC	PTELPPPSDRFE
ATCTGACCGTTTTGAAATCTATGGAGATGGCA	IYGDGNVKAY
ATGTCAAAGCATATTTTGTGACTGGGGAAGAC	FVTGEDIGTYT
ATTGGTACATACACCATGAAATCTGTGGACGA	MKSVDDPRTL
CCCACGAACTCTCAACAAGTCTCTGCATTTCA	NKSLHFRPPHN
GACCACCTCACAACTTTTTGTCATTGAATGAA	FLSLNEMANIW
ATGGCAAACATATGGGAGACCAAGATTCACA	ETKIHKTLPRA
AGACTCTGCCAAGGGCAGTCATTACCGAAGAT	VITEDDLLVIA
GATCTTCTGGTGATAGCAAAAGCTAATTTCAT	KANFMPSSIVA
GCCTTCAAGCATTGTGGCCGCACTCACCCATG	ALTHDIFINGC
ATATCTTCATCAATGGCTGCCAATTCAAGTTTC	QFKFPIKEPQD
CCATAAAAGAGCCACAAGATGTAGAAGCCTG	VEACELYPDFK
TGAGCTGTATCCAGATTTCAAATACACCACCA	YTTIEDFFSQYI
TTGAAGATTTTTTCAGCCAGTATATAATTTGA	I*

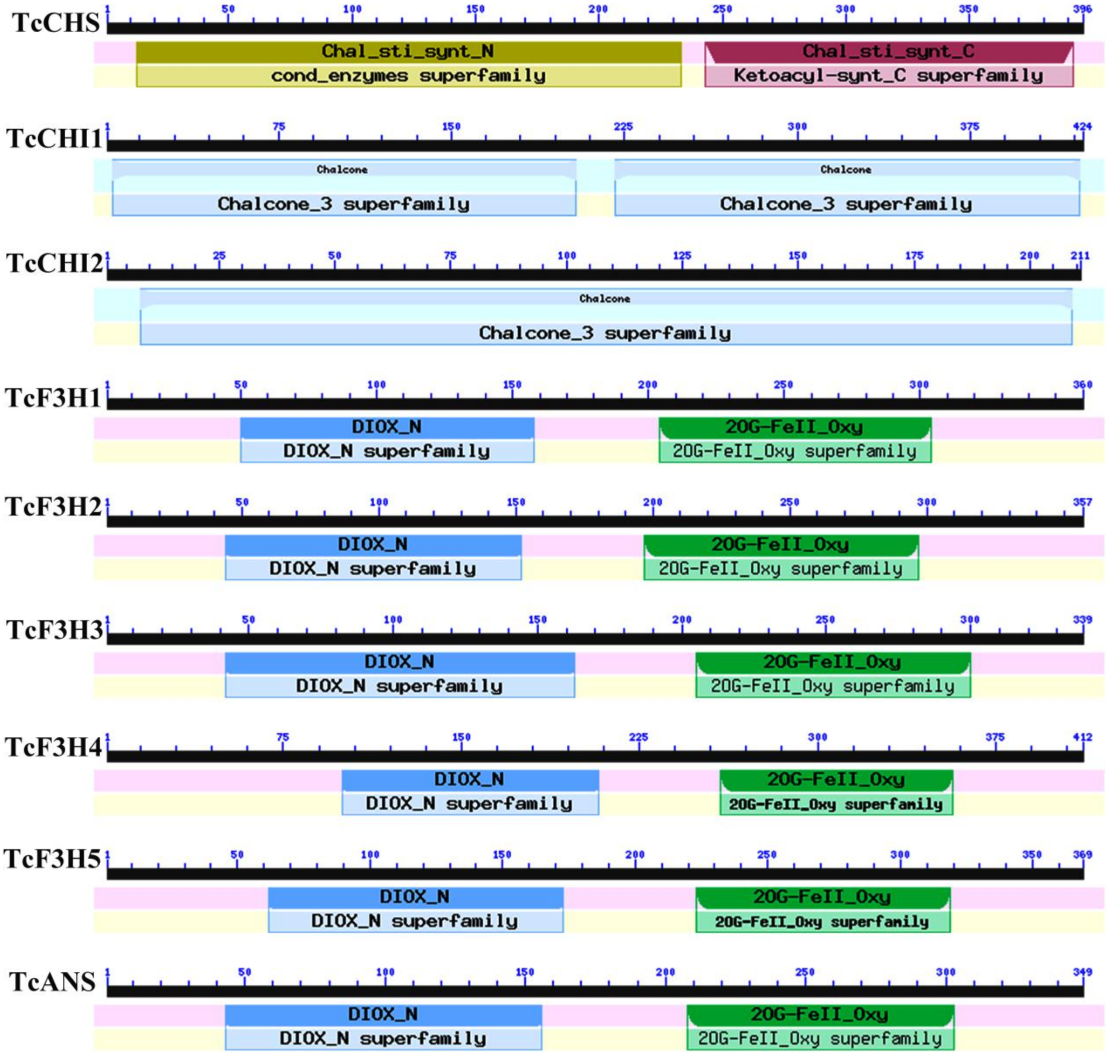

Fig S1. Conserved domains of TcCHS, TcCHI1-TcCHI2, TcF3H1-TcF3H5 and TcANS. Conserved domains were predicated by CD-search tool on NCBI (https://www.ncbi.nlm.nih.gov/Structure/cdd/wrpsb.cgi) against the Pfam v31.0 - 16709 PSSMs database and shown in colored boxes. Names of conserved domains were indicated below the boxes.

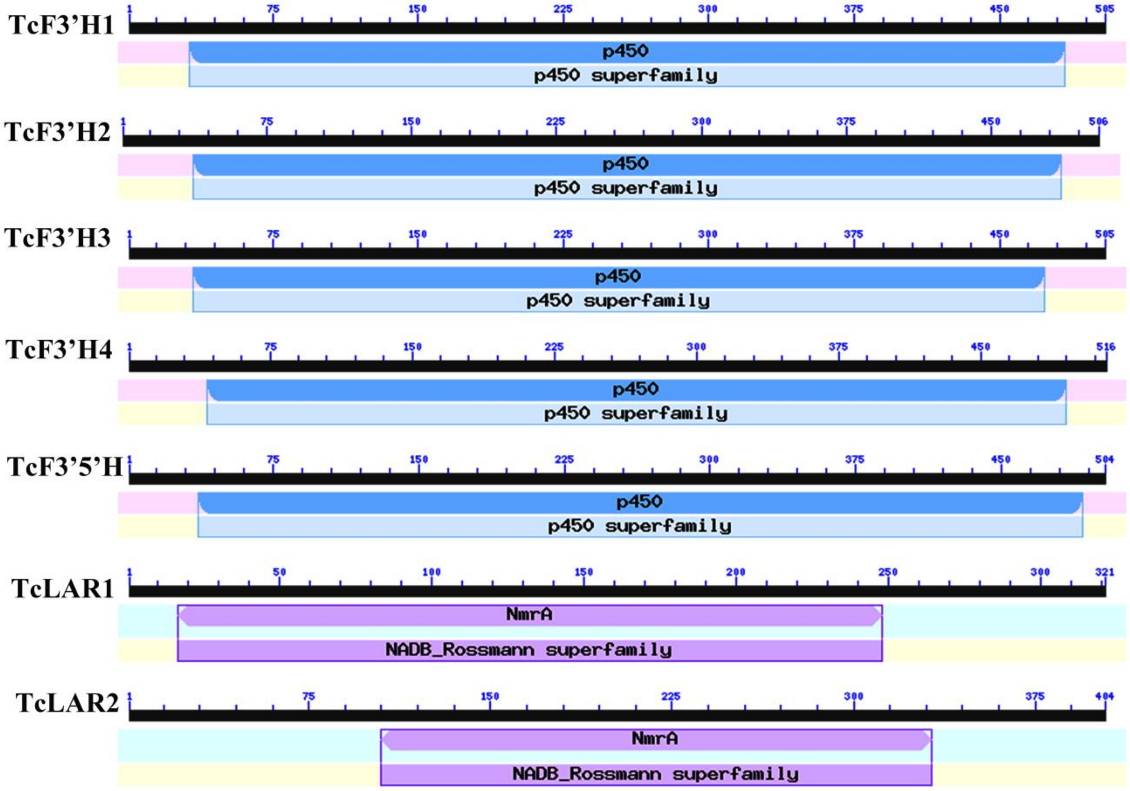

Fig S2. Conserved domains of TcF3'H1-TcF3'H4, TcF3'5'H and TcLAR1-TcLAR2. Conserved domains were predicated by CD-search tool on NCBI(https://www.ncbi.nlm.nih.gov/ Structure/cdd/wrpsb.cgi) against the Pfam v31.0 - 16709 PSSMs database and shown in colored boxes. Names of conserved domains were indicated below the boxes.

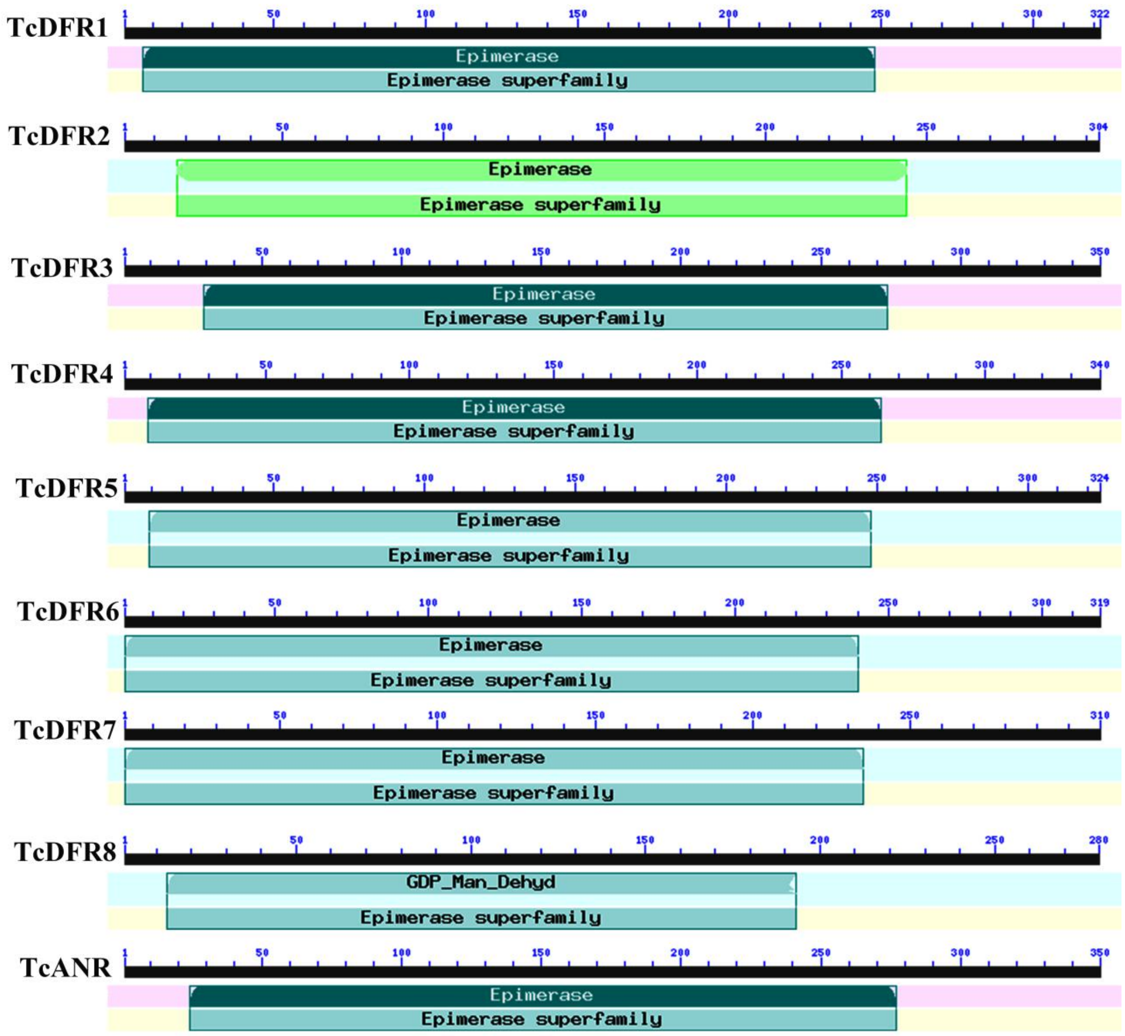

Fig S3. Conserved domains of TcDFR1-TcDFR8 and TcANR. Conserved domains were predicated by CD-search tool on NCBI(https://www.ncbi.nlm.nih.gov/ Structure/cdd/wrpsb.cgi) against the Pfam v31.0 - 16709 PSSMs database and shown in colored boxes. Names of conserved domains were indicated below the boxes.

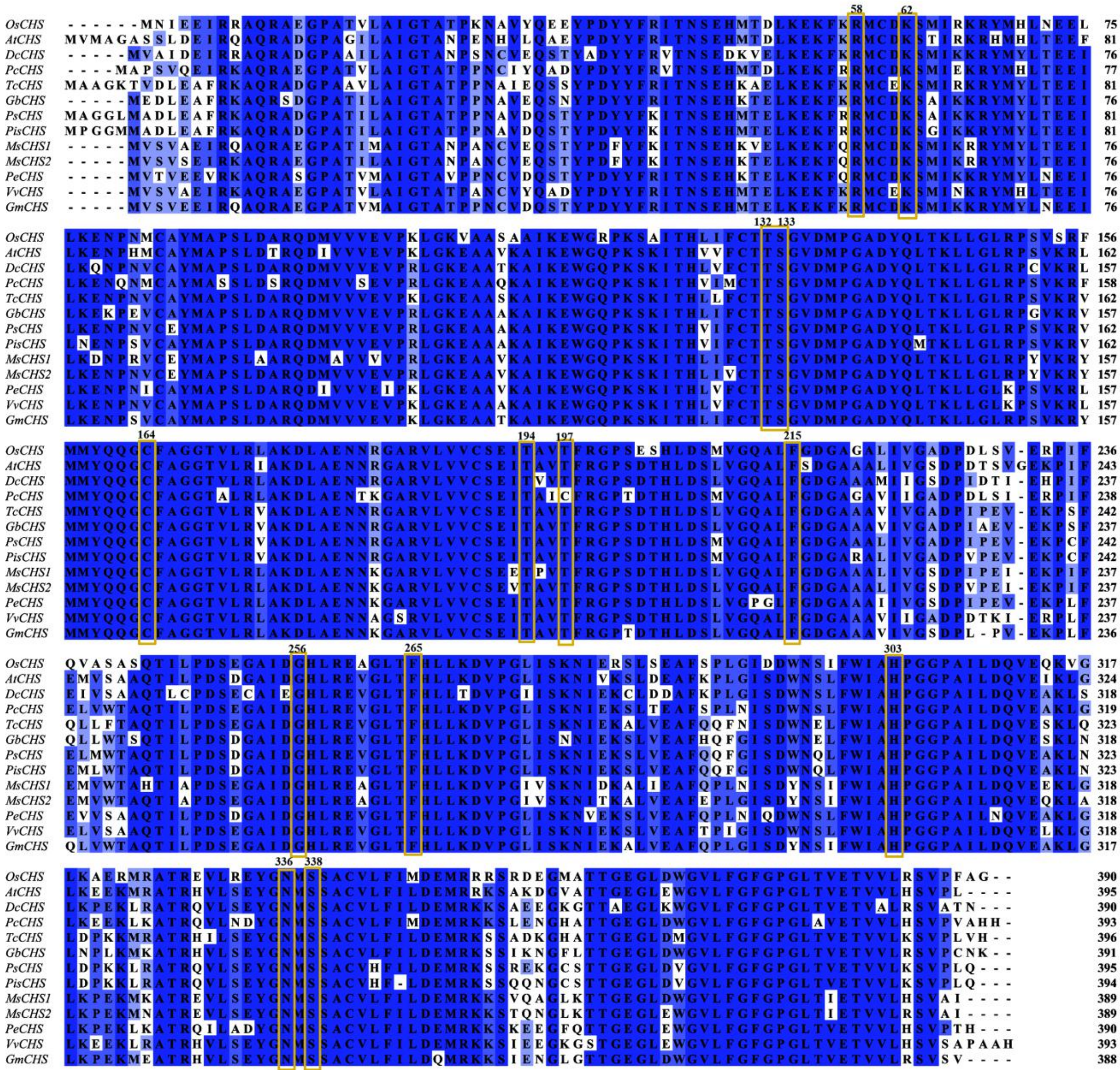

Fig S4. Multiple sequence alignment of TcCHS against CHSs from other plant spices. Identical sites were shown in blue background, enzyme active sites were shown in yellow boxes and the site numbers were above the boxes. The figure was produced using Clustal Omega tool on EMBL-EBI (https://www.ebi.ac.uk/Tools/msa/clustalo).

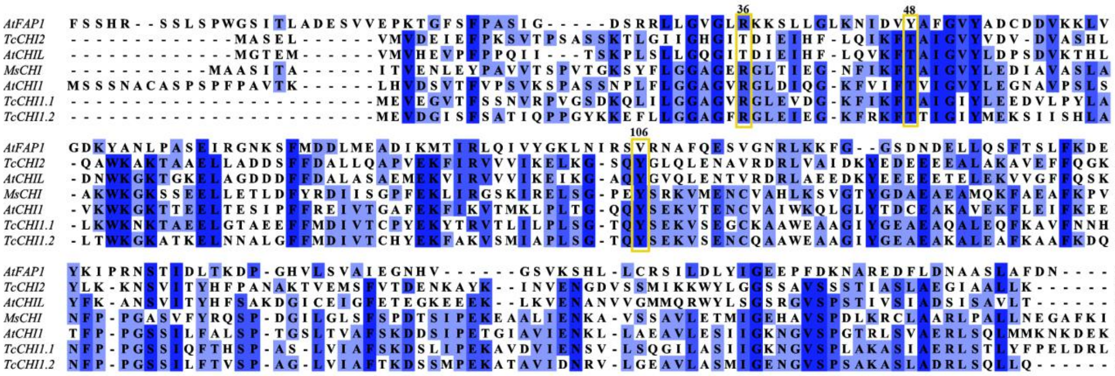

Fig S5. Multiple sequence alignment of TcCHI1-TcCHI2 against CHIs from other plant spices. Identical sites were shown in blue background, enzyme active sites were shown in yellow boxes and the site numbers were above the boxes. The figure was produced using Clustal Omega tool on EMBL-EBI (https://www.ebi.ac.uk/Tools/msa/clustalo).

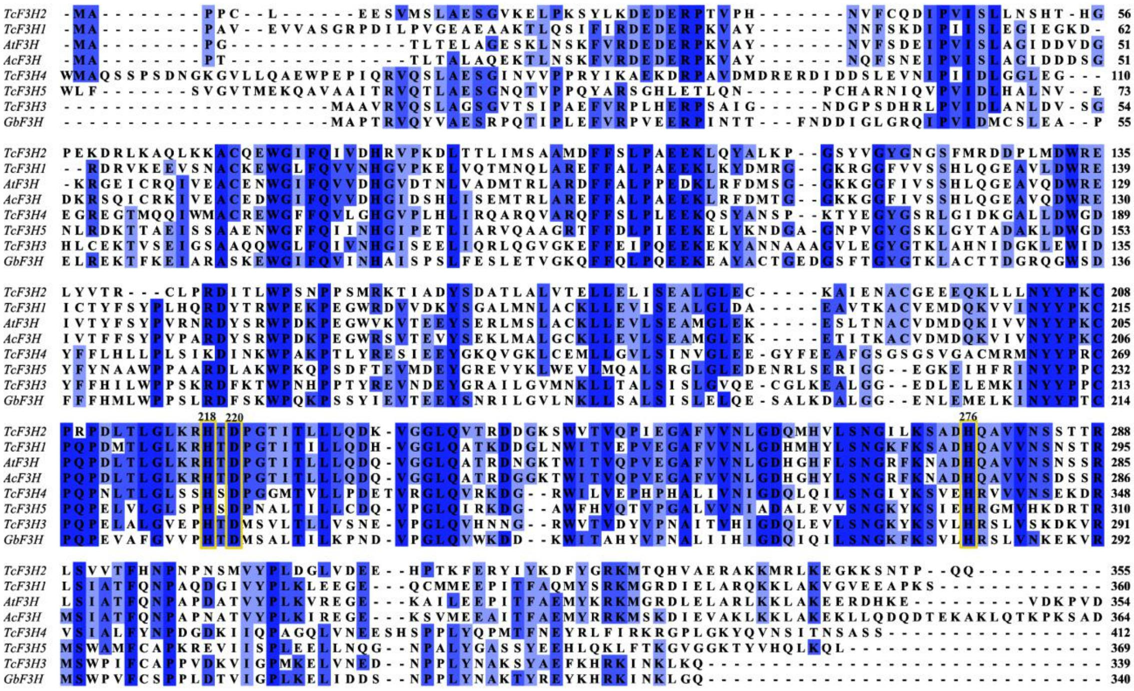

Fig S6. Multiple sequence alignment of TcF3H1-TcF3H5 against F3Hs from other plant spices. Identical sites were shown in blue background, enzyme active sites were shown in yellow boxes and the site numbers were above the boxes. The figure was produced using Clustal Omega tool on EMBL-EBI (https://www.ebi.ac.uk/Tools/msa/clustalo).

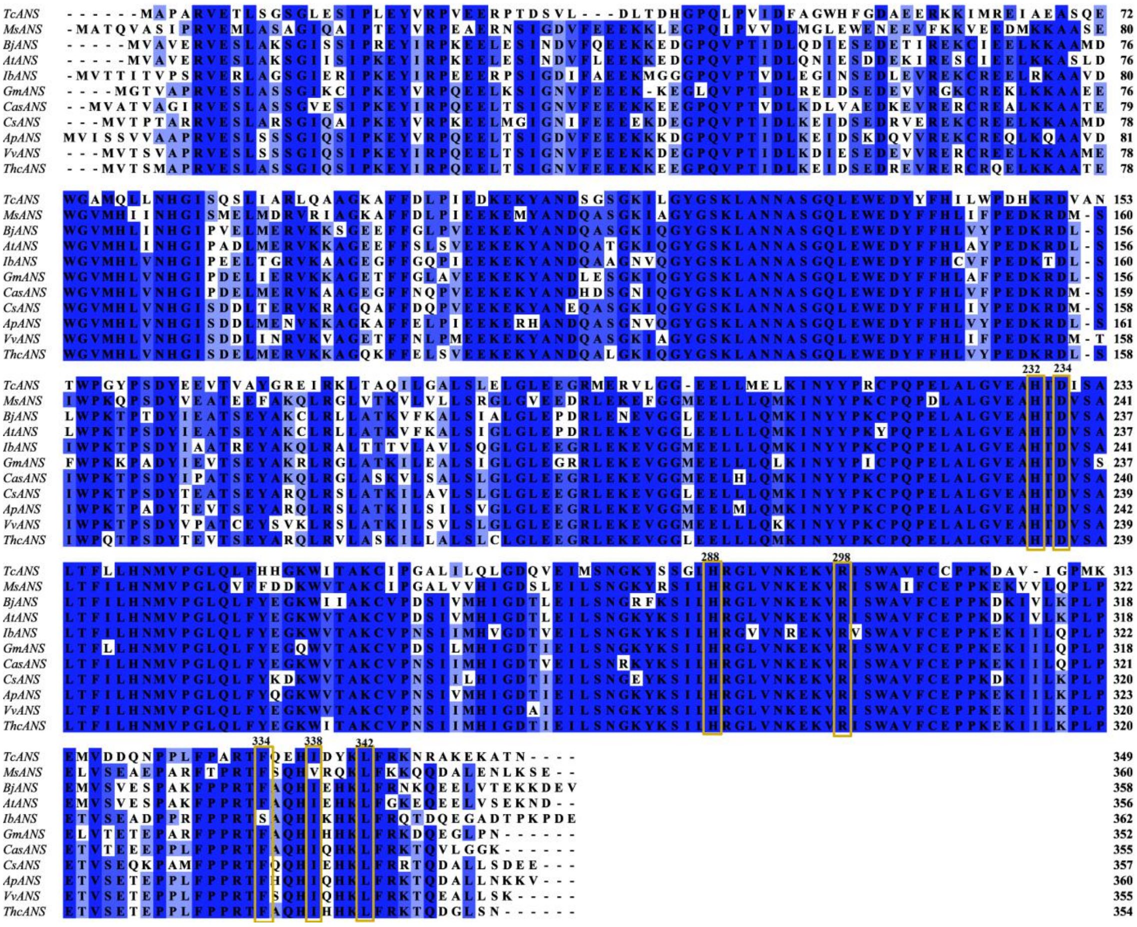

Fig S7. Multiple sequence alignment of TcANS against ANSs from other plant spices. Identical sites were shown in blue background, enzyme active sites were shown in yellow boxes and the site numbers were above the boxes. The figure was produced using Clustal Omega tool on EMBL-EBI (https://www.ebi.ac.uk/Tools/msa/clustalo).

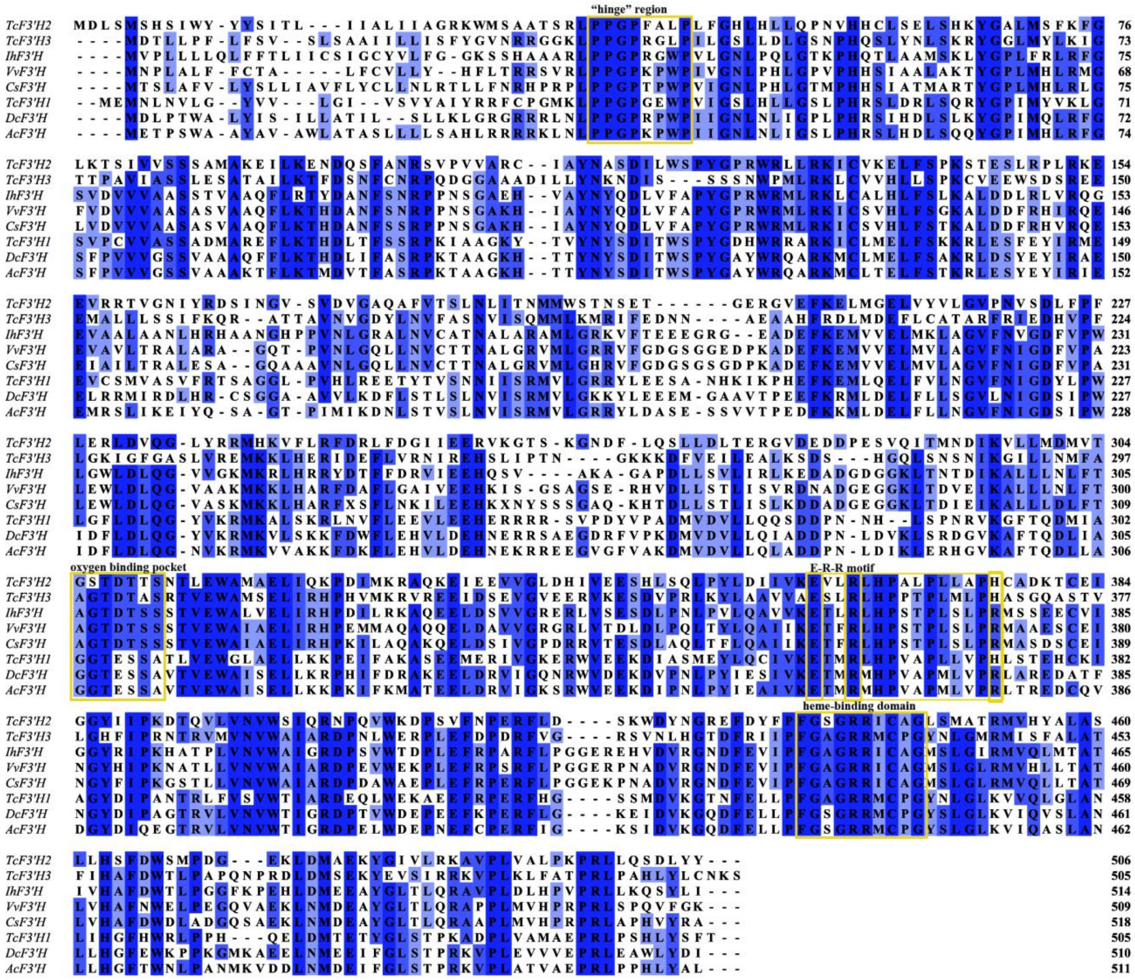

Fig S8. Multiple sequence alignment of TcF3'H1-TcF3'H4 against F3'Hs from other plant spices. Identical sites were shown in blue background, enzyme function domains were shown in yellow boxes and the domain names were above the boxes. The figure was produced using Clustal Omega tool on EMBL-EBI (https://www.ebi.ac.uk/Tools/msa/clustalo).

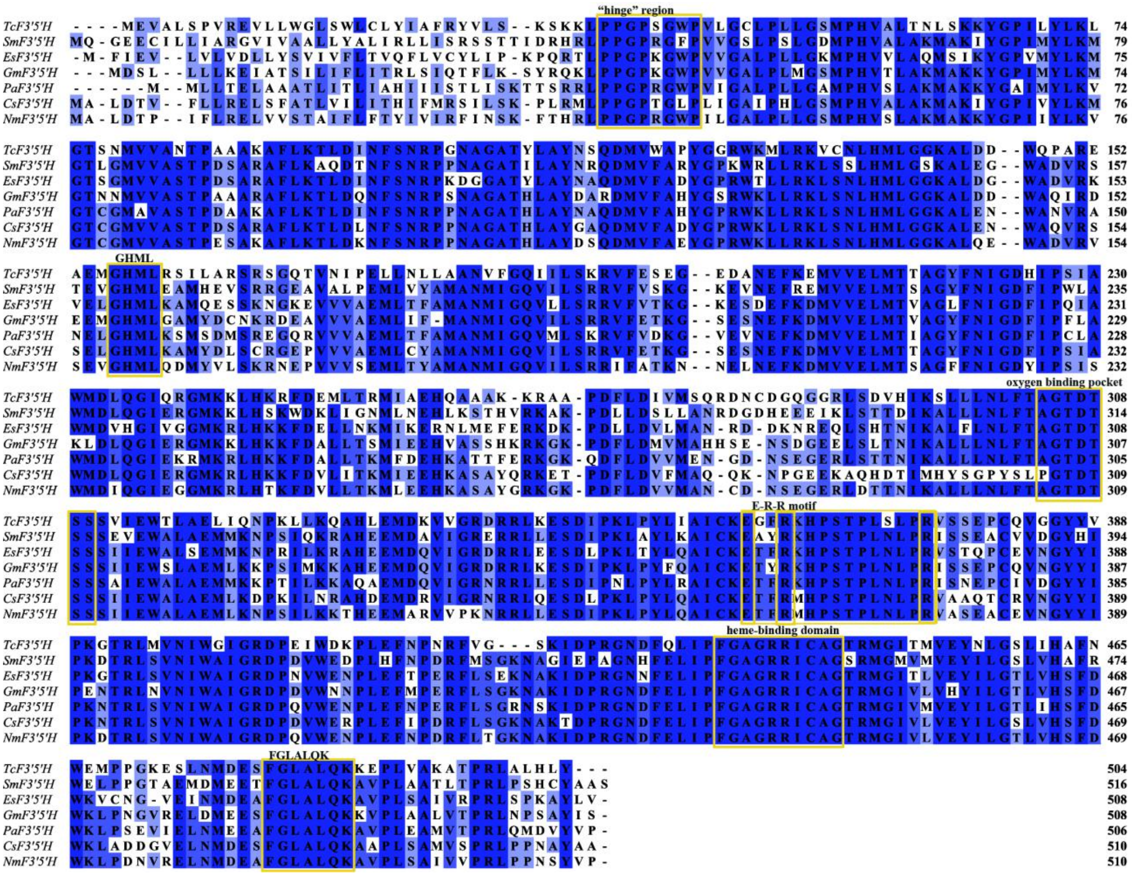

Fig S9. Multiple sequence alignment of TcF3'5'H against F3'5'Hs from other plant spices. Identical sites were shown in blue background, enzyme function domains were shown in yellow boxes and the domain names were above the boxes. The figure was produced using Clustal Omega tool on EMBL-EBI (https://www.ebi.ac.uk/Tools/msa/clustalo).

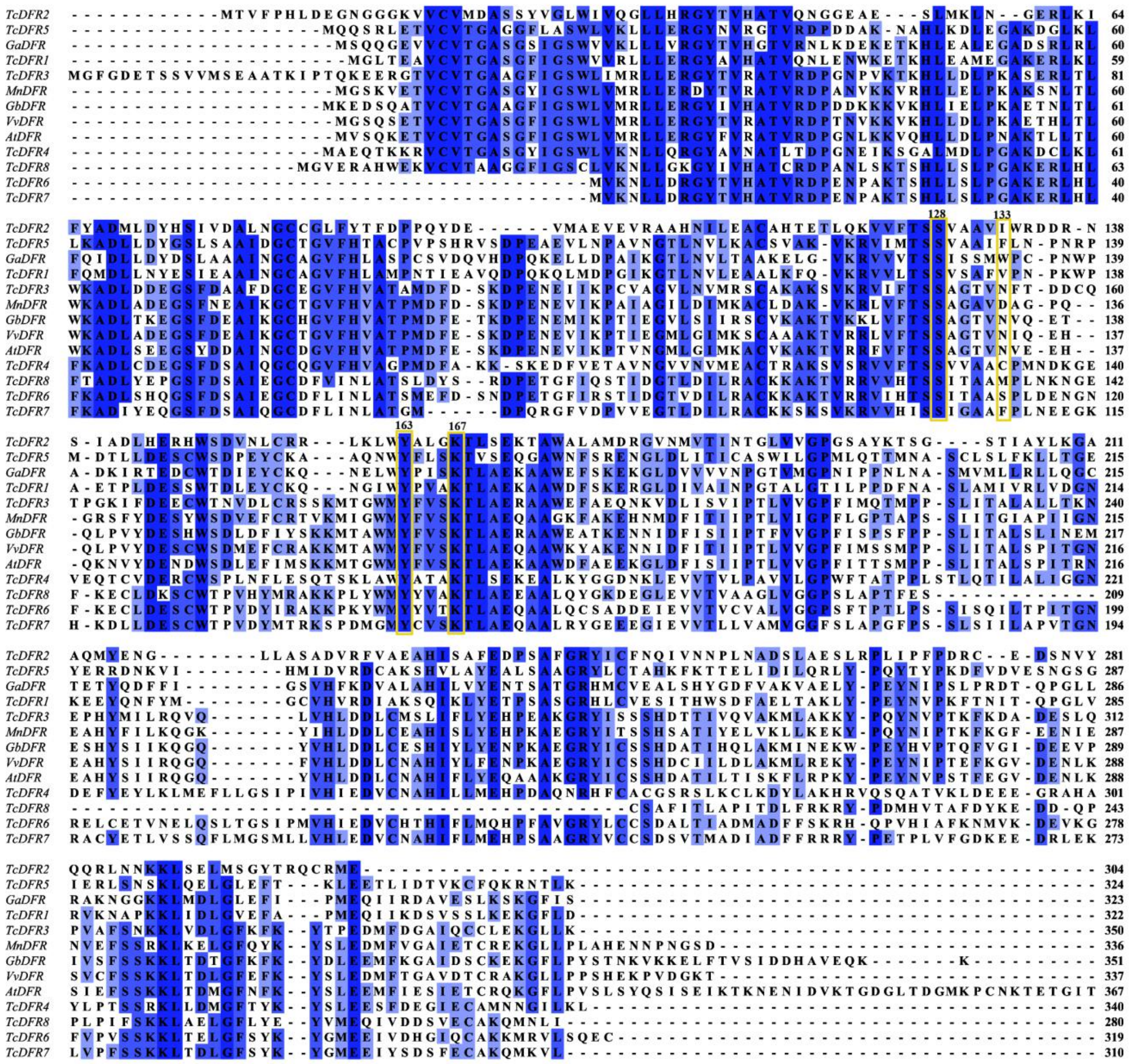

Fig S10. Multiple sequence alignment of TcDFR1-TcDFR8 against DFRs from other plant spices. Identical sites were shown in blue background, enzyme active sites were shown in yellow boxes and the site numbers were above the boxes. The figure was produced using Clustal Omega tool on EMBL-EBI (https://www.ebi.ac.uk/Tools/msa/clustalo).

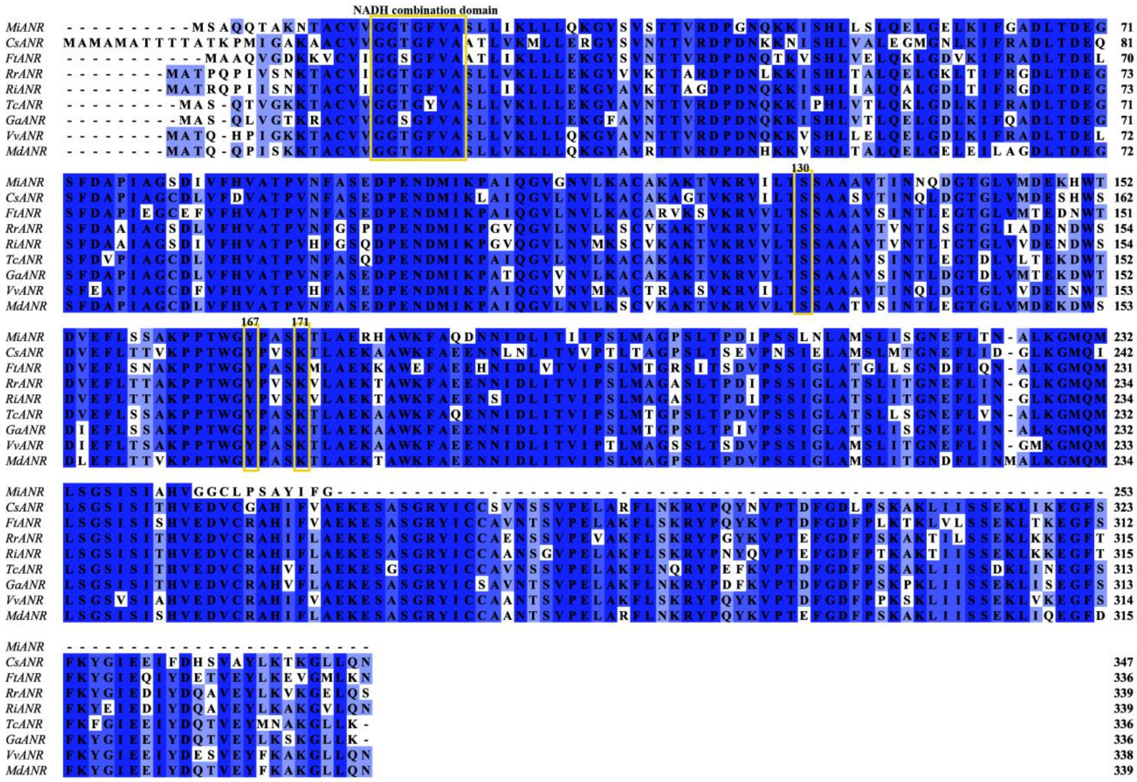

Fig S11. Multiple sequence alignment of TcANR against ANRs from other plant spices. Identical sites were shown in blue background, enzyme active sites were shown in yellow boxes and the site numbers are above the boxes, enzyme function domain was shown in yellow boxes and the domain name were above the boxe. The figure was produced using Clustal Omega tool on EMBL-EBI (https://www.ebi.ac.uk/Tools/msa/clustalo).

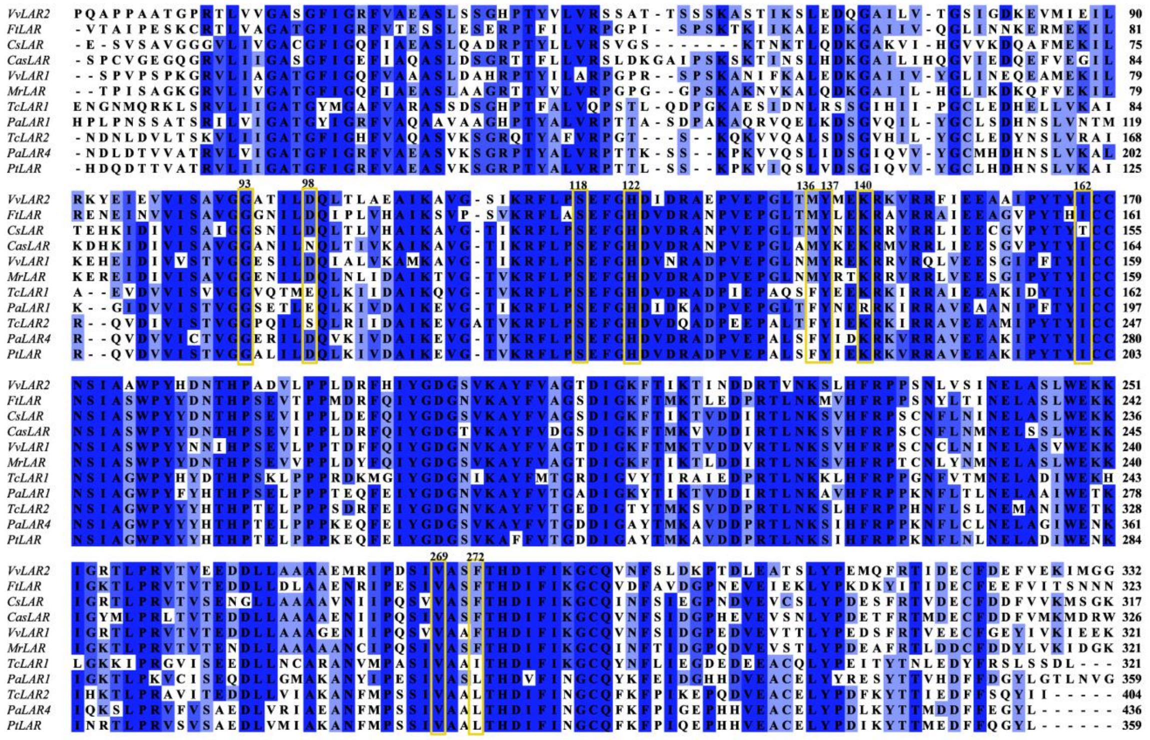

Fig S12. Multiple sequence alignment of TcLAR against LARs from other plant spices. Identical sites were shown in blue background, enzyme active sites were shown in yellow boxes and the site numbers were above the boxes. The figure was produced using Clustal Omega tool on EMBL-EBI (https://www.ebi.ac.uk/Tools/msa/clustalo).
